# TCN1 Drives Malignant Progression of Pancreatic Cancer Through STAT4-Mediated Transcriptional Activation of the DUOX2/ROS Signaling Axis

**DOI:** 10.3390/cancers17203300

**Published:** 2025-10-12

**Authors:** Zonglin Liu, Dongxue Ju, Ze Yu, Binru Zhang, Dongbo Xue, Yongwei Wang

**Affiliations:** 1 Department of Pancreatic and Biliary Surgery, The First Affiliated Hospital of Harbin Medical University, 23 Youzheng Street, Nangang District, Harbin 150001, China; 202201275@hrbmu.edu.cn (Z.L.); 2023021035@hrbmu.edu.cn (D.J.); 202401326@hrbmu.edu.cn (Z.Y.); 202201266@hrbmu.edu.cn (B.Z.); 2 Key Laboratory of Hepatosplenic Surgery, Ministry of Education, 23 Youzheng Street, Nangang District, Harbin 150001, China

**Keywords:** PDAC, TCN1, pancreatic cancer, STAT4, signaling axis

## Abstract

**Simple Summary:**

Pancreatic ductal adenocarcinoma is one of the deadliest cancers, and new biomarkers and treatment targets are urgently needed. We found that the protein transcobalamin 1 (TCN1) is markedly increased in pancreatic tumors and is linked to worse clinicopathologic features and poorer survival. In cell and mouse models, raising TCN1 enhanced tumor cell growth, movement, and invasion, while lowering TCN1 reduced these behaviors. Our data indicate that TCN1 is associated with the transcription factor STAT4, which in turn activates DUOX2 and elevates intracellular reactive oxygen species (ROS). Reducing STAT4 or DUOX2, or removing ROS with the antioxidants N-acetylcysteine or Tempol, weakened the aggressive effects driven by TCN1. These results suggest that the TCN1–STAT4–DUOX2/ROS pathway contributes to pancreatic cancer progression and that TCN1 may serve as a prognostic biomarker and potential therapeutic entry point.

**Abstract:**

Background: Pancreatic ductal adenocarcinoma (PDAC) is characterized by its aggressive clinical behavior and intricate microenvironment regulation, leading to dismal prognosis. Elucidating the molecular mechanisms underlying PDAC pathogenesis is crucial for developing improved therapeutic approaches. The functional significance and molecular basis of transcobalamin 1 (TCN1) in PDAC remain largely unexplored. Methods and Results: Through integrated analysis of TCGA and GTEx datasets combined with 80 clinical specimens, we identified significant TCN1 overexpression in PDAC, showing a positive association with tumor stage and negative associations with histological differentiation and overall survival. Functional investigations showed that TCN1 enhanced pancreatic cancer cell proliferation, migration, invasion, and epithelial–mesenchymal transition (EMT) in both in vitro and in vivo models. Mechanistically, TCN1 physically interacts with signal transducer and activator of transcription 4 (STAT4) to enhance its transcriptional activity. Chromatin immunoprecipitation (ChIP) assays showed that STAT4-mediated transcriptional activation of dual oxidase 2 (DUOX2) occurs through direct promoter binding. As a pivotal reactive oxygen species (ROS)-generating enzyme, DUOX2 overexpression elevates intracellular ROS levels, thereby promoting EMT progression and activating proliferation-related signaling cascades. Antioxidant treatment effectively abrogated TCN1-driven oncogenic phenotypes, establishing ROS as the critical downstream mediator. Conclusions: Collectively, our findings reveal a novel TCN1/STAT4/DUOX2 regulatory axis that exacerbates PDAC progression by remodeling redox homeostasis. This signaling cascade may serve as a prognostic biomarker and a potential therapeutic target for ROS-directed precision therapy in PDAC.

## 1. Introduction

Pancreatic ductal adenocarcinoma (PDAC), one of the most lethal malignancies worldwide, exhibits a 5-year survival rate of only 13% [1]. The aggressive clinical course primarily stems from aberrant tumor cell proliferation, metastatic potential, high tumor heterogeneity, and a sophisticated microenvironmental regulatory network that collectively drive therapeutic resistance and dismal prognosis [2,3,4,5,6]. Although emerging strategies targeting tumor microenvironment modulation and immune checkpoint blockade-based combination therapies have shown partial clinical benefits, overall survival improvements remain marginal [7,8,9]. Therefore, systematic elucidation of the molecular mechanisms underlying PDAC progression, identification of reliable prognostic biomarkers, and discovery of actionable therapeutic targets constitute critical unmet needs in current PDAC management.

Reactive oxygen species (ROS) serve as pivotal drivers of malignant progression in multiple cancer types [10,11,12]. Within the tumor microenvironment, sustained oncogenic activation coupled with persistent NADPH oxidase (NOX) family activity induces ROS accumulation, which orchestrates multifaceted mechanisms to promote aggressive tumor phenotypes. ROS-mediated activation of the PI3K/Akt/mTOR and MAPK signaling pathways upregulates SNAIL and MMP-2/9 expression, thereby enhancing tumor cell proliferation and motility. Moreover, ROS potentiate angiogenesis through VEGF signaling activation [13,14,15,16]. Notably, ROS directly induce DNA base oxidation and impair damage repair mechanisms, consequently exacerbating the carcinogenic mutation burden [17,18]. As a critical ROS-generating enzyme within the NOX family, dual oxidase 2 (DUOX2) has been implicated in tumor pathogenesis. Emerging evidence has revealed marked DUOX2 upregulation across various solid malignancies, including colorectal and gastric cancers, where its expression correlates with adverse clinical outcomes [19,20,21,22,23]. However, the regulatory mechanisms governing the DUOX2/ROS axis in pancreatic cancer and its causal relationship with malignant transformation remain poorly characterized.

Transcobalamin 1 (TCN1), an essential protein mediating vitamin B12 transport, has been classically associated with cellular metabolic regulation [24,25]. Emerging evidence has identified its non-canonical role in transcriptional reprogramming, particularly in driving colorectal cancer progression [26]. In this study, we established a significant association between elevated TCN1 expression and poor prognosis in patients with PDAC. Functional characterization revealed that TCN1 enhances pancreatic cancer cell proliferation, migration, invasion, and epithelial–mesenchymal transition (EMT) in both in vitro and in vivo models, thereby accelerating tumor progression. Mechanistic investigations revealed that TCN1 physically interacts with signal transducer and activator of transcription 4 (STAT4) to modulate its transcriptional activity, which subsequently regulates DUOX2 expression. This regulatory cascade disrupts redox homeostasis and drives PDAC malignancy. Importantly, genetic or pharmacological inhibition of ROS effectively reversed the TCN1-mediated oncogenic effects, confirming ROS as the terminal executor of this signaling axis. Collectively, our study delineates the TCN1/STAT4/DUOX2/ROS regulatory network in PDAC pathogenesis. These findings provide a novel prognostic biomarker as well as uncover targetable therapeutic vulnerabilities for precision interventions in pancreatic cancer management.

## 2. Materials and Methods

### Patient Cohort and Specimen Collection

Eighty paired PDAC tissues and adjacent non-tumor tissues were retrospectively collected from patients undergoing curative pancreatectomy at the First Affiliated Hospital of Harbin Medical University (Harbin, China) between 2015 and 2024. Inclusion criteria: histologically confirmed PDAC, no neoadjuvant therapy, complete clinicopathologic and follow-up data. Exclusion criteria: prior malignancy, perioperative death, or inadequate tissue. Tumor stage and grade followed AJCC (8th ed.) and WHO criteria. Baseline clinicopathologic characteristics and their associations with TCN1 expression are summarized in Table 1. Fresh samples were snap-frozen in liquid nitrogen and stored at −80 °C; portions were formalin-fixed and paraffin-embedded (FFPE) for IHC. Written informed consent was obtained from all participants. This study was approved by the institutional ethics committee (Approval No. 2025130). Additional method details are provided in Doc S1.

## 3. Results

### 3.1. Expression Characteristics and Clinical Significance of TCN1 in Pancreatic Cancer

**Figure 1:**
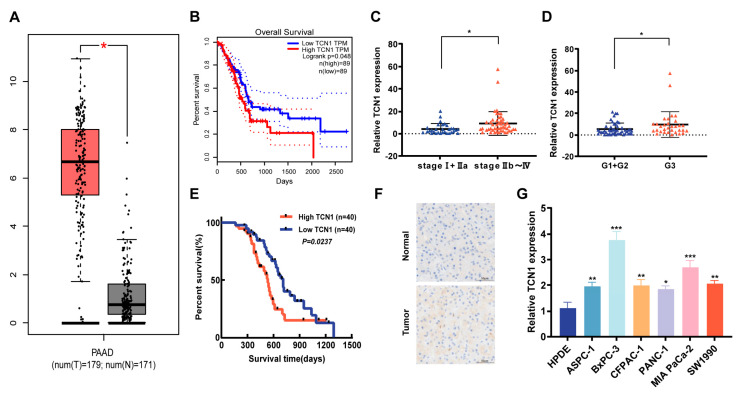
Expression characteristics and clinical significance of TCN1 in pancreatic cancer. (**A**) Relative expression levels of TCN1 in pancreatic cancer tissues versus adjacent normal tissues from the TCGA and GTEx databases. (**B**) Overall survival of patients stratified into high-TCN1 and low-TCN1 expression groups based on TCGA and GTEx data. (**C**,**D**) qRT-PCR analysis of TCN1 expression in PDAC tissues categorized by TNM stage and pathological grade. (**E**) Overall survival analysis of pancreatic cancer patients with high versus low TCN1 expression in an in-house cohort. (**F**) Immunohistochemical (IHC) staining validating TCN1 expression in PDAC tissues compared to that of adjacent normal tissues. (**G**) qRT-PCR quantification of TCN1 expression levels across pancreatic cancer cell lines. Three independent experiments were performed. Data is displayed as the mean ± SD. ns: no significance; * *p* < 0.05; ** *p* < 0.01; *** *p* < 0.001.

Although TCN1 has been implicated in promoting progression across multiple malignancies, its precise role in PDAC remains elusive. Our preliminary analysis of the TCGA and GTEx databases revealed significantly elevated TCN1 expression in PDAC tissues compared to that in normal pancreatic tissues (Figure 1A), revealing its potential involvement in PDAC tumorigenesis. Survival analysis revealed that patients with high TCN1 expression exhibited significantly shorter overall survival than those with low expression (Figure 1B), indicating a strong correlation between TCN1 levels and poor prognosis. To validate these findings, we analyzed 80 clinical PDAC samples. The quantitative reverse transcription-polymerase chain reaction (qRT-PCR) results showed markedly higher TCN1 mRNA levels in advanced-stage (IIb–IV) tumors than in early stage (I–IIa) counterparts (Figure 1C), with poorly differentiated tumors displaying significantly elevated TCN1 expression compared to that of well-differentiated lesions (Figure 1D). Kaplan–Meier analysis of this cohort confirmed that high TCN1 expression predicted reduced 5-year survival rates (Figure 1E), consistent with the TCGA/GTEx findings. Immunohistochemical (IHC) staining further revealed the predominant cytoplasmic localization of TCN1 protein in PDAC tissues, with significantly higher positivity rates than in adjacent normal tissues (Figure 1F). Evaluation of TCN1 expression across five PDAC cell lines (ASPC-1, BxPC-3, CFPAC-1, MIA PaCa-2, and SW1990) versus normal pancreatic ductal epithelial (HPDE) cells showed universal TCN1 upregulation in malignant cells (Figure 1G). Notably, BxPC-3 cells showed the highest TCN1 expression, whereas PANC-1 cells exhibited the lowest levels, reflecting cellular heterogeneity that paralleled clinical differentiation-grade correlations. Collectively, these findings establish TCN1 as a consistently overexpressed molecular feature in PDAC, with its expression levels strongly associated with tumor stage, histological differentiation, and patient survival. This positions TCN1 as a potential biomarker for monitoring PDAC progression and for therapeutic stratification.

### 3.2. Regulatory Role of TCN1 in Pancreatic Cancer Cell Proliferation

**Figure 2:**
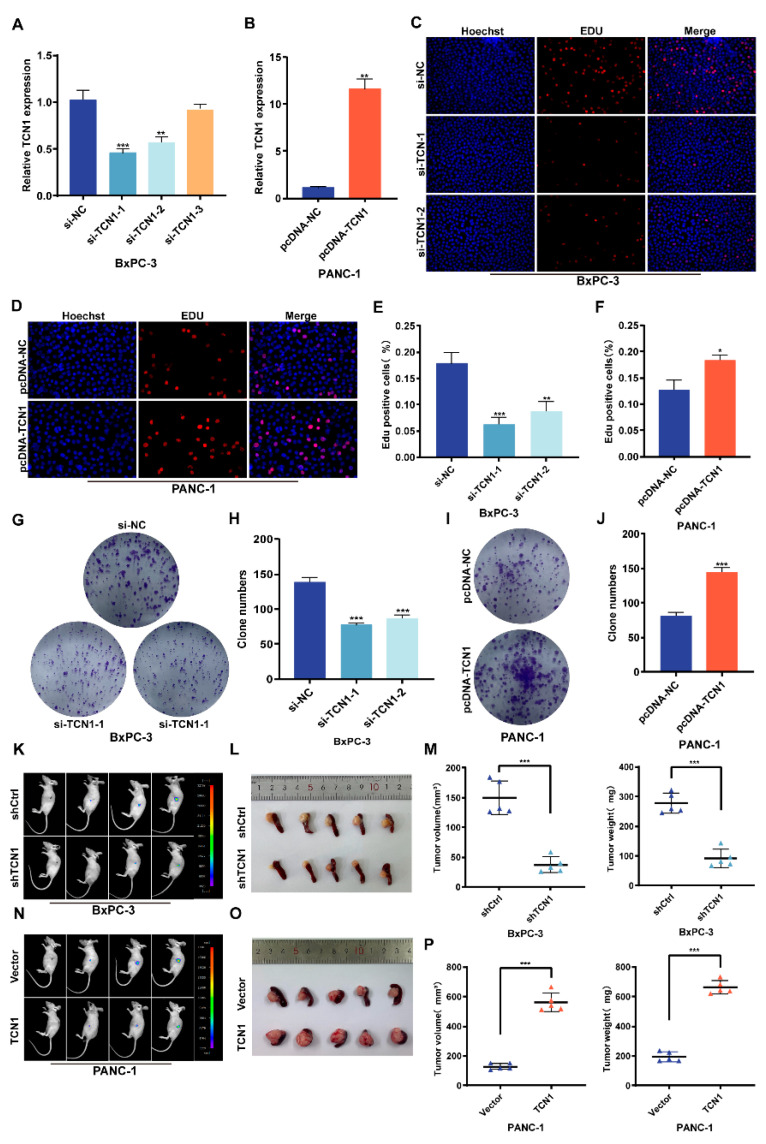
Regulatory role of TCN1 in pancreatic cancer cell proliferation. (**A**,**B**) qRT-PCR validation of TCN1 knockdown and overexpression in BxPC-3 and PANC-1 cells transfected with three distinct siRNAs or a pcDNA-TCN1 plasmid. (**C**–**F**) EdU proliferation assays assessing proliferative activity in BxPC-3 (knockdown) and PANC-1 (overexpression) cells following TCN1 modulation. (**G**–**J**) Colony formation assays evaluating the impact of TCN1 knockdown or overexpression on cell proliferation. (**K**–**P**) Representative bioluminescence imaging of orthotopic tumors in mice at days 7, 14, 21, and 28. All mice were sacrificed on day 28, and pancreatic primary tumors were excised, photographed, and analyzed for tumor volume and weight. Three independent experiments were performed. Data is displayed as the mean ± SD. ns: no significance; * *p* < 0.05; ** *p* < 0.01; *** *p* < 0.001.

To further elucidate the biological significance of TCN1 in pancreatic cancer progression, we selected BxPC-3 (high TCN1 expression) and PANC-1 (low TCN1 expression) cell lines based on their differential gene expression profiles. A genetic modulation system was established using siRNA (si-TCN1) for knockdown and pcDNA-TCN1 overexpression plasmid for upregulation. qRT-PCR confirmed significant suppression of TCN1 mRNA levels in the knockdown group and a marked elevation in the overexpression group (Figure 2A,B). EdU proliferation assays revealed that TCN1 knockdown significantly inhibited DNA synthesis activity in BxPC-3 cells, leading to a reduced proliferative capacity, whereas TCN1 overexpression markedly enhanced the proliferative activity of PANC-1 cells (Figure 2C–F). To further validate the impact of TCN on self-renewal ability, colony formation assays showed that TCN1 knockdown substantially decreased the number of BxPC-3 cell colonies, whereas TCN1 overexpression significantly increased the colony-forming efficiency of PANC-1 cells (Figure 2G–J). Furthermore, to investigate the role of TCN1 in pancreatic cancer progression, we established stable TCN1-knockdown (BxPC-3) and TCN1-overexpressing (PANC-1) cell lines through lentiviral infection, with successful modulation confirmed using qRT-PCR (Supplementary Figure S1A,B). These cells were then implanted into the pancreas of nude mice to generate orthotopic tumor model. Over 4 weeks of observation, tumors in the TCN1-knockdown group exhibited significantly slower growth rates than those in the control group (Figure 2K). Post-dissection quantitative analyses further revealed that tumors with TCN1 knockdown had markedly reduced mass and volume compared to those in the control group (Figure 2L,M). Conversely, TCN1-overexpressing orthotopic tumors displayed accelerated growth (Figure 2N) and significantly increased tumor mass and volume (Figure 2O,P). Collectively, these findings indicate that TCN1 acts as a key regulatory molecule in pancreatic cancer, sustaining tumor growth by promoting proliferative activity.

### 3.3. TCN1 Promotes Migration, Invasion, and EMT Progression in Pancreatic Cancer

**Figure 3:**
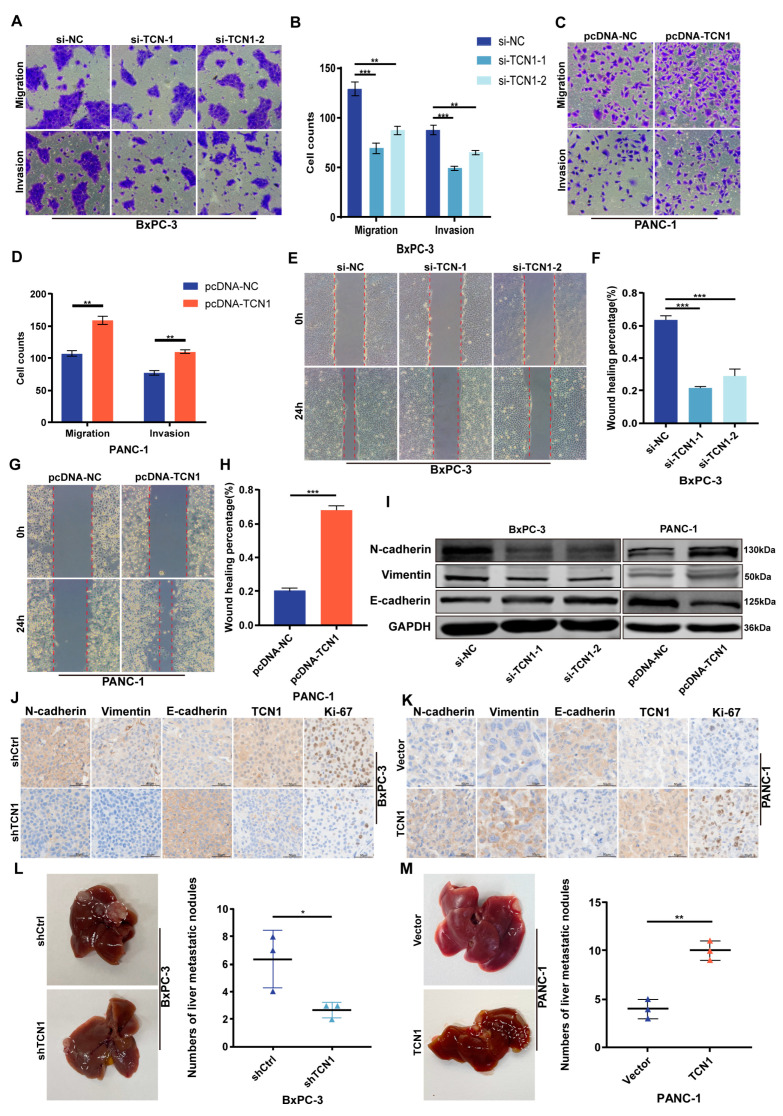
TCN1 promotes migration, invasion, and EMT progression in pancreatic cancer. (**A**–**D**) Transwell invasion assays (original magnification 20×) assessing the impact of TCN1 knockdown or overexpression on the invasive and metastatic capacities of BxPC-3 and PANC-1 cells. (**E**–**H**) Wound-healing assays evaluating the migratory ability of pancreatic cancer cells following TCN1 knockdown or overexpression. (**I**) Western blot analysis of N-cadherin, vimentin, and E-cadherin expression in BxPC-3 and PANC-1 cells after TCN1 modulation. (**J**,**K**) IHC staining (original magnification 20×; scale bar, 400 μm) of paraffin-embedded orthotopic tumor sections for N-cadherin, vimentin, E-cadherin, TCN1, and Ki-67 expression. (**L**,**M**) Representative images of hepatic metastasis models established with TCN1-knockdown or -overexpressing cells, with macroscopic quantification of visible metastatic nodules. Three independent experiments were performed. Data is displayed as the mean ± SD. ns: no significance; * *p* < 0.05; ** *p* < 0.01; *** *p* < 0.001.

To further investigate the role of TCN1 in metastasis-related phenotypes, Transwell migration and invasion assays revealed that TCN1 knockdown significantly suppressed the transmembrane capacity of BxPC-3 cells, whereas TCN1-overexpressing PANC-1 cells exhibited a marked increase in transmigration (Figure 3A–D). Wound-healing assays further confirmed that TCN1 knockdown delayed scratch closure in BxPC-3 cells, whereas TCN1 overexpression enhanced the migratory ability of PANC-1 cells (Figure 3E–H). To elucidate the molecular mechanisms underlying TCN1-mediated malignant behavior, Western blot analysis showed that TCN1 knockdown in BxPC-3 cells upregulated the epithelial marker E-cadherin and downregulated the mesenchymal markers N-cadherin and vimentin. Conversely, TCN1 overexpression in PANC-1 cells suppressed E-cadherin expression, while significantly elevating N-cadherin and vimentin levels (Figure 3I; Supplementary Figure S2A). IHC of orthotopic tumor tissues from nude mice corroborated these findings: TCN1-knockdown tumors exhibited reduced N-cadherin and vimentin expression, but increased E-cadherin expression. In addition, Ki-67, a key proliferation marker, was downregulated in TCN1-knockdown tumors and upregulated in TCN1-overexpressing tumors (Figure 3J,K; Supplementary Figure S2B,C), indicating that TCN1 may enhance metastatic potential by activating the EMT pathway. To directly evaluate the impact of TCN1 on tumor cell metastasis in vivo, we established a nude mouse hepatic metastasis model. Consistent with our hypothesis, TCN1-knockdown mice displayed significantly fewer liver tumor nodules than the controls, whereas TCN1-overexpressing mice exhibited increased metastatic foci (Figure 3L,M). These results indicate that TCN1 promotes pancreatic cancer cell proliferation, migration, invasion, and EMT in vitro and in vivo.

### 3.4. DUOX2 Is Transcriptionally Regulated by TCN1 and Drives Malignant Phenotypes in Pancreatic Cancer

**Figure 4:**
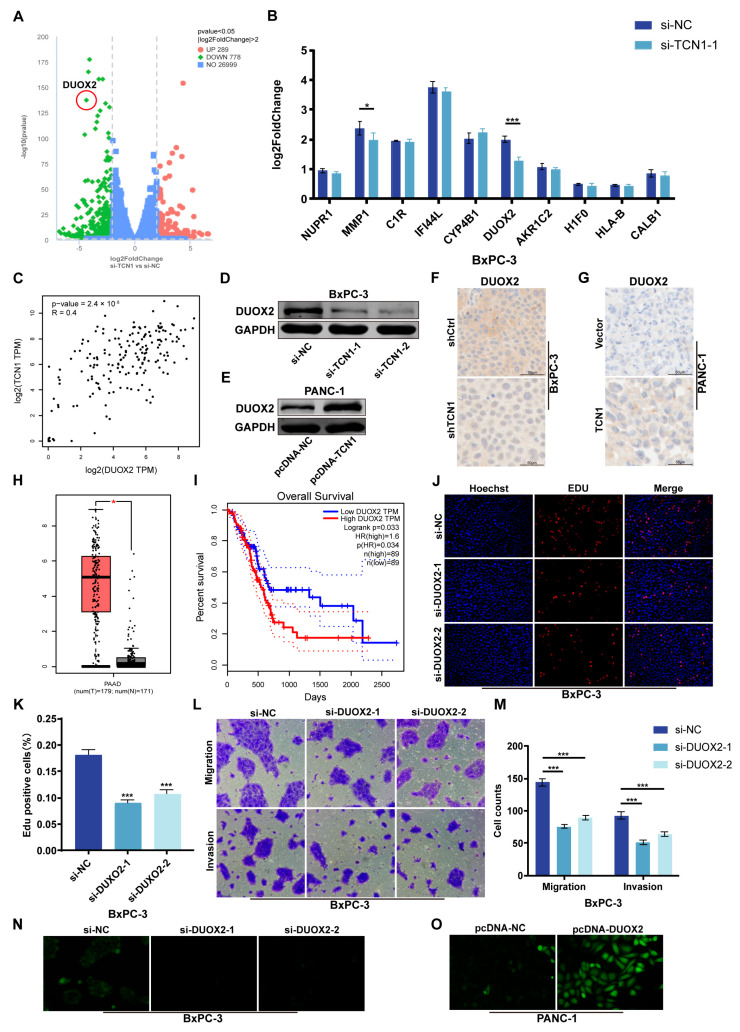
Transcriptional regulation of DUOX2 by TCN1 and its oncogenic role in pancreatic cancer malignancy. (**A**) Volcano plot illustrating differentially expressed genes following TCN1 knockdown. (**B**) qRT-PCR validation of the relative expression levels of the top 10 differentially expressed genes. (**C**) Correlation between TCN1 and DUOX2 expressions in the GEPIA database. (**D**,**E**) Western blot analysis of DUOX2 protein levels in BxPC-3 and PANC-1 cells after TCN1 knockdown or overexpression. (**F**,**G**) IHC staining of DUOX2 expression in pancreatic orthotopic tumor tissues following TCN1 modulation. (**H**) Relative expression of DUOX2 in pancreatic cancer versus adjacent normal tissues from TCGA and GTEx databases. (**I**) Overall survival of patients stratified into high-DUOX2 and low-DUOX2 expression groups (TCGA and GTEx data). (**J**,**K**) EdU proliferation assays assessing the proliferative capacity of BxPC-3 cells after DUOX2 knockdown. (**L**,**M**) Transwell migration/invasion assays evaluating metastatic and invasive abilities of BxPC-3 cells with DUOX2 knockdown. (**N**,**O**) Measurement of intracellular ROS levels in BxPC-3 and PANC-1 cells following DUOX2 knockdown or overexpression. Three independent experiments were performed. Data is displayed as the mean ± SD. ns: no significance; * *p* < 0.05; *** *p* < 0.001.

To further explore the regulatory mechanism of TCN1 in pancreatic cancer malignancy, RNA sequencing (RNA-seq) was performed in TCN1-knockdown BxPC-3 cells. Differential expression analysis (|log2FC| ≥ 2, *p* < 0.05) identified DUOX2 as a top candidate gene significantly downregulated upon TCN1 suppression (Figure 4A). qRT-PCR validation of the top 10 differentially expressed genes confirmed that DUOX2 mRNA levels were markedly reduced in TCN1-knockdown cells (Figure 4B). Analysis of pancreatic cancer tissues from the GEPIA database revealed a strong positive correlation between TCN1 and DUOX2 expression, further supporting their regulatory relationship (Figure 4C). The Western blotting results showed that TCN1 knockdown significantly decreased DUOX2 protein levels, whereas TCN1 overexpression significantly upregulated DUOX2 expression (Figure 4D,E; Supplementary Figure S3A). IHC of tumor tissues mirrored these results, showing reduced DUOX2 staining in TCN1-knockdown tumors and elevated staining in TCN1-overexpressing tumors (Figure 4F,G; Supplementary Figure S3B,C), indicating the transcriptional and translational regulation of DUOX2 by TCN1. Analysis of the TCGA and GTEx datasets revealed that DUOX2 expression was significantly higher in pancreatic cancer tissues than in normal tissues, and its elevated expression correlated with shorter overall patient survival, showing that DUOX2 is a potential oncogenic driver (Figure 4H,I). To investigate the functional role of DUOX2, siRNA (si-DUOX2) and pcDNA-DUOX2 plasmids were used to establish knockdown and over-expression systems in BxPC-3 and PANC-1 cells, respectively, with efficiency confirmed by qRT-PCR (Supplementary Figure S3D,E). EdU proliferation and colony formation assays showed that DUOX2 knockdown suppressed pancreatic cancer cell proliferation, whereas DUOX2 overexpression enhanced these malignant phenotypes (Figure 4J,K; Supplementary Figure S4A–F). Transwell migration/invasion and wound-healing assays further revealed that DUOX2 knockdown attenuated tumor cell migratory and invasive capacities, whereas DUOX2 overexpression robustly promoted these behaviors (Figure 4L,M; Supplementary Figure S5A–F), confirming the critical role of DUOX2 in pancreatic cancer metastasis. As a member of the dual oxidase family, DUOX2 activation is associated with ROS generation. Experiments showed that DUOX2 knockdown reduced intracellular ROS levels, whereas DUOX2 overexpression significantly increased ROS production (Figure 4N,O). In addition, Western blot analysis showed that DUOX2 knockdown downregulated mesenchymal markers (N-cadherin and vimentin) and upregulated the epithelial marker E-cadherin, with opposite trends observed in DUOX2-overexpressing cells (Supplementary Figure S5G,H). These findings indicate that DUOX2 regulates both ROS generation and invasive phenotypes, with a potential causal interplay between these mechanisms.

### 3.5. TCN1 Promotes Pancreatic Cancer Malignancy by Activating DUOX2

**Figure 5:**
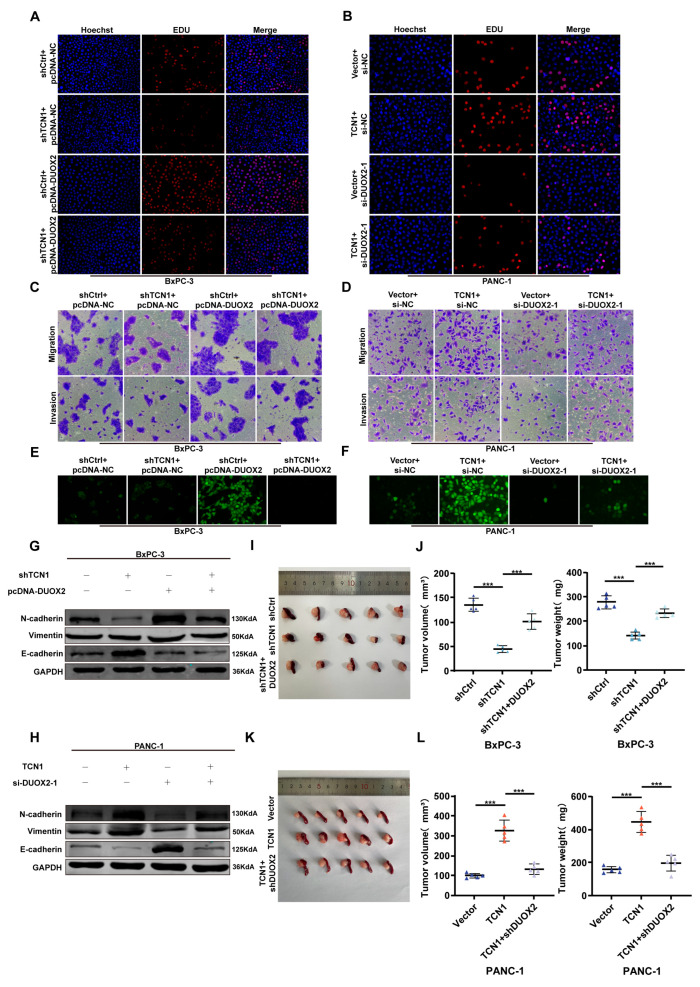
TCN1 promotes pancreatic cancer malignancy by activating DUOX2. (**A**,**B**) EdU proliferation assays evaluating the rescue effect of DUOX2 knockdown or overexpression on TCN1-mediated changes in cell proliferation. (**C**,**D**) Transwell migration/invasion assays assessing the restoration of TCN1-driven migratory and invasive capacities by DUOX2 knockdown or overexpression. (**E**,**F**) ROS detection assays revealing the rescue of TCN1-induced ROS generation through DUOX2 modulation. (**G**,**H**) Western blot analysis of EMT-related markers (N-cadherin, vimentin, and E-cadherin) showing the reversal of TCN1-mediated EMT progression by DUOX2 knockdown or overexpression. (**I**–**L**) In vivo validation of the rescue effect of DUOX2 modulation on TCN1-driven tumor growth (volume and mass) in orthotopic models. Three independent experiments were performed. Data is displayed as the mean ± SD. ns: no significance; *** *p* < 0.001.

In a previous study, we established the functional roles of TCN1 and DUOX2 in pancreatic cancer progression and their regulatory relationships. However, whether TCN1 drives pancreatic cancer progression through DUOX2-dependent signaling remains unverified. To address this, we conducted rescue experiments between TCN1 and DUOX2. EdU proliferation assays showed that TCN1 knockdown significantly suppressed proliferative activity in BxPC-3 cells, whereas exogenous DUOX2 overexpression reversed this inhibition (Figure 5A; Supplementary Figure S6A). Conversely, TCN1 overexpression in PANC-1 cells markedly enhanced proliferation, but this effect was partially abrogated by DUOX2 knockdown (Figure 5B; Supplementary Figure S6B). Colony formation assays further revealed that DUOX2 overexpression partially restored the proliferation suppressed by TCN1 knockdown in BxPC-3 cells (Supplementary Figure S6C,D), whereas DUOX2 knockdown significantly attenuated the pro-proliferative effect of TCN1 overexpression in PANC-1 cells (Supplementary Figure S6E,F). Transwell migration/invasion and wound-healing assays revealed that TCN1 knockdown in BxPC-3 cells significantly inhibited their migratory and invasive capacities, whereas DUOX2 overexpression partially rescued these metastatic phenotypes (Figure 5C; Supplementary Figure S7A,C,D). In contrast, TCN1-overexpressing PANC-1 cells exhibited enhanced migration and invasion, which were completely blocked by DUOX2 knockdown (Figure 5D; Supplementary Figure S7B,E,F). Subsequent analysis of intracellular ROS levels showed that TCN1 knockdown in BxPC-3 cells significantly suppressed ROS generation, whereas DUOX2 overexpression restored ROS production (Figure 5E). Conversely, TCN1-overexpressing PANC-1 cells displayed elevated ROS levels, which returned to baseline upon DUOX2 knockdown (Figure 5F). Western blotting further showed that TCN1 knockdown in BxPC-3 cells upregulated the epithelial marker E-cadherin and downregulated the mesenchymal markers (N-cadherin and vimentin), whereas DUOX2 overexpression reversed these EMT-related protein changes (Figure 5G; Supplementary Figure S7G). In PANC-1 cells, TCN1 overexpression induced EMT through DUOX2 activation, an effect that was abolished by DUOX2 knockdown (Figure 5H; Supplementary Figure S7H). To determine whether DUOX2 mediates the biological functions of TCN in vivo, we combined TCN1 knockdown with DUOX2 overexpression in orthotopic tumors. DUOX2 restoration fully reversed the tumor volume reduction caused by TCN1 knockdown (Figure 5I,J). Conversely, co-knockdown of DUOX2 in TCN1-overexpressing tumors significantly suppressed TCN1-driven tumor growth (Figure 5K,L). Collectively, these findings indicate that TCN1 promotes pancreatic cancer malignancy by activating DUOX2-dependent signaling, which drives ROS generation and facilitates aggressive tumor behavior.

### 3.6. TCN1 Binds and Regulates Transcription Factor STAT4 to Promote DUOX2 Transcription

**Figure 6:**
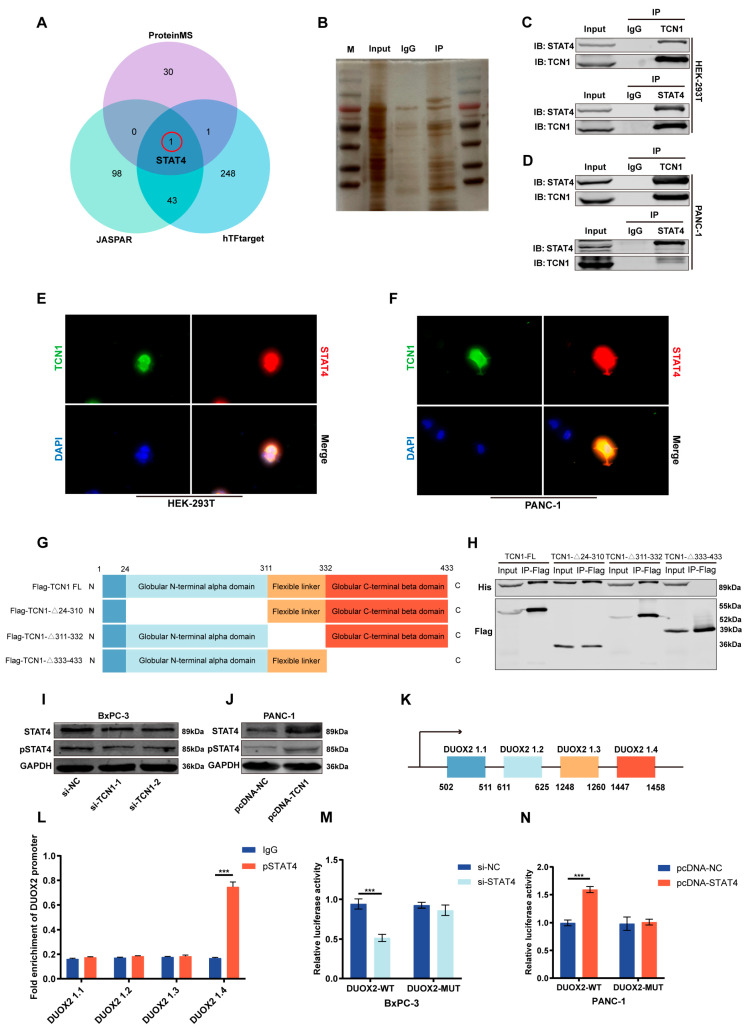
TCN1 binds and regulates transcription factor STAT4 to promote DUOX2 transcription. (**A**) Intersection of proteomic results with JASPAR and hTFtarget databases. (**B**) Silver staining identifying TCN1-interacting proteins in HEK-293T cells. (**C**,**D**) Co-immunoprecipitation analysis of TCN1–STAT4 interaction in HEK-293T and PANC-1 cell lines. (**E**,**F**) Representative immunofluorescence images showing TCN1–STAT4 co-localization in HEK-293T and PANC-1 cells (scale bars: 20 μm). (**G**) Schematic of full-length TCN1 truncation into distinct functional domains. (**H**) Western blot identification of STAT4-binding domains in TCN1 using domain-specific constructs. (**I**,**J**) Western blot analysis of STAT4 and phosphorylated STAT4 (pSTAT4) expression under TCN1 knockdown or overexpression conditions. (**K**) Predicted STAT4-binding motifs within the DUOX2 promoter region. (**L**) qRT-PCR quantification of STAT4 occupancy at DUOX2 promoter sites in PANC-1 cells (IgG as negative control). (**M**,**N**) Relative luciferase activity of wild-type and mutant (MUT) DUOX2 reporter plasmids in STAT4-knockdown/overexpressing BxPC-3 and PANC-1 cells. Three independent experiments were performed. Data is displayed as the mean ± SD. ns: no significance; *** *p* < 0.001.

To elucidate the molecular mechanism through which TCN1 regulates DUOX2, we first performed mass spectrometry to identify potential interacting proteins in TCN1-overexpressing cells. Concurrently, we systematically screened candidate transcriptional regulators of DUOX2 using prediction databases, such as hTFtarget. By integrating the mass spectrometry dataset with multi-source transcription factor predictions and using a cross-comparison strategy, we identified signal transducer and activator of transcription 4 (STAT4) as the critical hub molecule in the TCN1-DUOX2 regulatory network (Figure 6A). Silver staining confirmed the presence of precipitated proteins (Figure 6B). To validate the direct interaction between TCN1 and STAT4, co-immunoprecipitation (Co-IP) assays in 293T cells showed that TCN1 pulldown specifically captured STAT4, and reciprocally, STAT4 pulldown efficiently enriched TCN1 (Figure 6C). To confirm the universality of this interaction, we repeated the assay in PANC-1 cells, consistently observing bidirectional binding between TCN1 and STAT4 (Figure 6D). Immunofluorescence (IF) co-localization analysis further revealed significant spatial co-localization of TCN1 and STAT4 in both 293T and PANC-1 cells (Figure 6E,F). To map the functional domains of TCN1 required for STAT4 binding, we constructed three TCN1 truncation mutants: deletion of the N-terminal α-domain (24–310 aa), flexible linker region (311–332 aa), or C-terminal β-domain (333–433 aa) (Figure 6G). Co-IP assays showed that only deletion of the C-terminal β-domain abolished TCN1–STAT4 binding (Figure 6H), indicating that this region is critical for their interactions. Notably, qRT-PCR showed that, regardless of TCN1 silencing or overexpression, STAT4 mRNA levels exhibited no significant change(Supplementary Figure S8A,B). These observations suggest that TCN1 primarily modulates STAT4 at post-transcriptional/post-translational levels. Consistently, TCN1 knockdown markedly reduced STAT4 and phospho-STAT4 protein abundance, whereas TCN1 overexpression increased both (Figure 6I,6J; Supplementary Figure S8C). STAT4 was predicted to bind to four putative sites within the DUOX2 promoter (Figure 6K). ChIP assays confirmed that STAT4 specifically binds to the DUOX2 promoter region (DUOX2 1.4) (Figure 6L). To assess the functional relevance of this binding, we generated wild-type and DUOX2 1.4-mutated luciferase reporter constructs. STAT4 knockdown or overexpression significantly suppressed or enhanced luciferase activity in the wild-type promoter, whereas mutation of DUOX2 1.4 abolished these effects (Figure 6M,N).

### 3.7. TCN1/STAT4/DUOX2 Axis Drives Pancreatic Cancer Progression Through ROS

**Figure 7:**
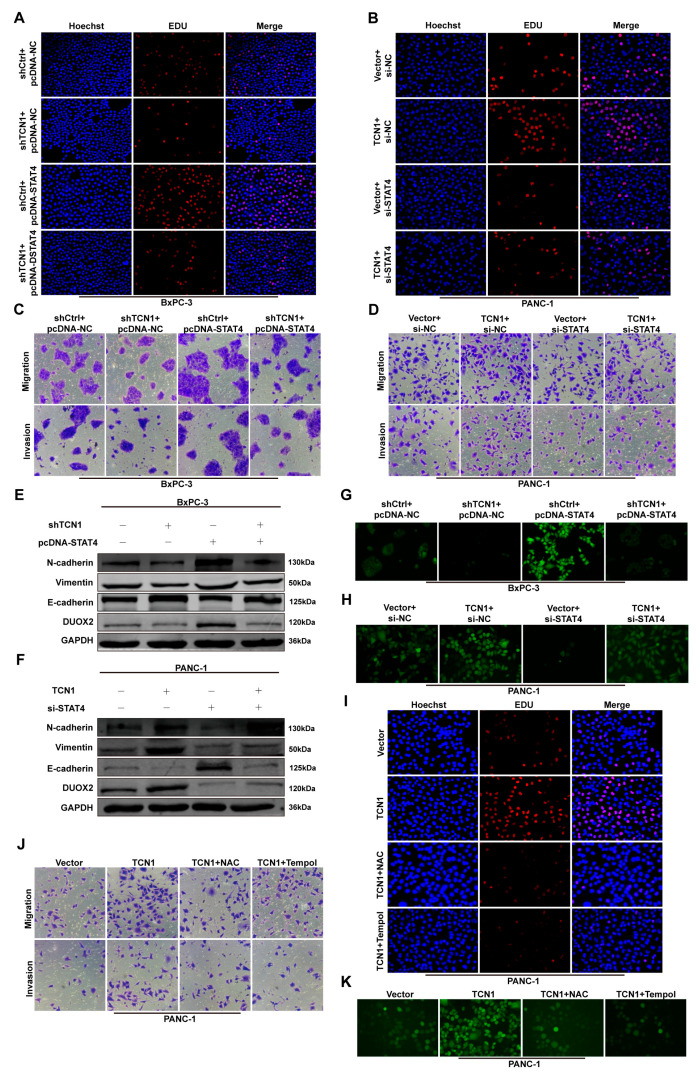
TCN1/STAT4/DUOX2 axis drives pancreatic cancer progression through ROS. (**A**,**B**) EdU proliferation assays evaluating the rescue effect of STAT4 knockdown or overexpression on TCN1-mediated changes in cell proliferation. (**C**,**D**) Transwell migration/invasion assays assessing the restoration of TCN1-driven migratory and invasive capacities by STAT4 modulation. (**E**,**F**) Western blot analysis of EMT markers (E-cadherin, N-cadherin, and vimentin) showing the reversal of TCN1-induced EMT progression through STAT4 knockdown or overexpression. (**G**,**H**) ROS detection assays showing the rescue of TCN1-dependent ROS generation through STAT4 modulation. (**I**) EdU proliferation assays evaluating the reversal of TCN1-driven proliferative enhancement by the antioxidant N-acetylcysteine (NAC) and 4-Hydroxy-TEMPO (Tempol). (**J**) Transwell migration/invasion assays demonstrate that N-acetylcysteine (NAC) and Tempol suppress TCN1-induced migratory and invasive capacities. (**K**) ROS detection assays confirm that NAC and Tempol counteract the TCN1-dependent increase in ROS. Three independent experiments were performed. Data is displayed as the mean ± SD. ns: no significance.

To further delineate the mechanism through which the TCN1/STAT4/DUOX2 axis promotes pancreatic cancer malignancy, we systematically performed functional rescue experiments in BxPC-3 and PANC-1 cells. EdU proliferation and colony formation assays revealed that TCN1 knockdown significantly suppressed pancreatic cancer cell proliferation, whereas STAT4 overexpression rescued this inhibitory effect. Conversely, STAT4 knockdown in TCN1-overexpressing cells markedly attenuated TCN1-driven proliferation (Figure 7A,B; Supplementary Figure S9A–F). Similarly, Transwell migration/invasion and wound-healing assays showed that TCN1 knockdown impaired the migratory and invasive capacities of the cells, which were restored by STAT4 overexpression. Conversely, TCN1 overexpression combined with STAT4 knockdown reversed the pro-metastatic effects (Figure 7C,D; Supplementary Figure S10A–F). Western blot analysis showed that TCN1 knockdown downregulated DUOX2 protein levels and suppressed EMT marker expression, whereas STAT4 overexpression fully restored DUOX2 expression and reversed EMT inhibition. Conversely, TCN1 overexpression upregulated DUOX2 expression and promoted EMT, and this effect was abolished by STAT4 knockdown (Figure 7E,F; Supplementary Figure S10G,H), indicating that TCN1 regulates DUOX2 through STAT4 to drive EMT-mediated tumor aggressiveness. ROS assays revealed that TCN1 knockdown suppressed intracellular ROS generation, which was rescued by STAT4 overexpression. Conversely, TCN1 overexpression significantly increased ROS levels, and this effect was reversed by STAT4 knockdown (Figure 7G,H). To directly validate ROS as a functional mediator of the TCN1/STAT4/DUOX2 axis, we treated TCN1-overexpressing PANC-1 cells with the antioxidants N-acetylcysteine (NAC) and 4-Hydroxy-TEMPO (Tempol). Treatment with either NAC or Tempol significantly reversed TCN1-driven enhancements in proliferation (Figure 7I; Supplementary Figure S11A–C), migration/invasion (Figure 7J; Supplementary Figure S11D–F), and ROS production (Figure 7K), thereby confirming ROS as a critical downstream effector of this axis.

## 4. Discussion

PDAC, one of the most aggressive malignancies within the digestive system, exhibits a striking concordance between its incidence and mortality rates. Global cancer epidemiological data have revealed a persistent increase in the disease burden of PDAC, with mortality increasing by 1.5% annually from 2013 to 2022 [1]. The clinical use of carbohydrate antigen 19-9 (CA19-9), the primary diagnostic biomarker, is limited by its sensitivity of only 75–85%. Although late recurrence rates gradually decline within 1–2 years postoperatively, the overall recurrence rate remains alarmingly high, exceeding 80% within 5 years post-surgery [27]. These findings highlight critical scientific bottlenecks in the current diagnostic and therapeutic frameworks, spanning both molecular diagnostic accuracy and therapeutic intervention efficacy. Consequently, elucidating the molecular regulatory networks underlying PDAC pathogenesis and developing clinically actionable molecular biomarkers and innovative therapeutic targets have emerged as central scientific priorities in pancreatic cancer research.

As a specific transporter of vitamin B12, TCN1 regulates metabolic homeostasis by mediating the transmembrane transport of vitamin B12. Vitamin B12, a critical coenzyme for methylation reactions and a central regulator of malonyl-CoA metabolism, plays essential roles in one-carbon metabolism and lipid biosynthesis [28,29]. Although traditional studies have focused on the involvement of TCN1 in tumorigenesis through these canonical metabolic pathways, its non-canonical mechanisms within the tumor microenvironment remain unclear. Consistent with the oncogenic roles of TCN1 in colorectal cancer, cholangiocarcinoma, and other gastrointestinal malignancies [30,31], we observed significant upregulation of TCN1 in PDAC tissues. Its expression levels correlated negatively with tumor stage, differentiation grade, and patient survival, findings validated in both public databases and our in-house cohort. Functional assays further showed that TCN1 drives malignant phenotypes in pancreatic cancer by promoting proliferation, migration, and EMT, a pivotal process enabling tumor invasion and metastasis [32,33]. These findings were corroborated in vivo, with orthotopic tumor models exhibiting high TCN1 expression and accelerated tumor growth, further supporting the role of TCN1 as an oncogenic driver in PDAC progression.

Protein–protein interactions (PPI) represent a core mechanism governing cellular processes and are closely associated with tumor initiation, progression, invasion, and drug resistance [34]. Further mechanistic studies revealed that TCN1 interacts with STAT4 at the molecular level and is spatially co-localized. The C-terminal β-domain of TCN1 (333–433 aa) was identified as the critical region mediating its binding to STAT4. Characterization of this structural feature provides a theoretical foundation for molecular interventions targeting the TCN1–STAT4 interaction. In parallel, chromatin immunoprecipitation (ChIP) and dual-luciferase reporter assays indicated that STAT4 occupies the DUOX2 promoter and drives its transcription, thereby linking this axis to ROS generation and EMT phenotypes. Taken together, these lines of evidence indicate that the TCN1/STAT4/DUOX2/ROS cascade constitutes a tumor cell-autonomous regulatory pathway distinct from the modes of STAT4 activation observed in immune or stromal cells [35], thereby highlighting a PDAC-specific, cell type–restricted regulatory milieu and potential actionable points of intervention.

STAT4, a pivotal component of the JAK/STAT4 signaling pathway, contributing to the shaping of the immune microenvironment and regulating transcriptional programs within tumor cells. [36,37,38]. In PDAC cells, our results indicate that STAT4 participates in the transcriptional regulation of DUOX2 and corresponds to changes in intracellular ROS levels. Perturbations in TCN1 levels are accompanied by concomitant alterations in DUOX2 expression and ROS readouts. Notably, STAT4 mRNA remains largely unchanged upon either TCN1 knockdown or overexpression, consistent with regulation occurring at post-transcriptional or post-translational layers. Together with shifts in total and phosphorylated STAT4, these observations suggest potential involvement of protein stability, site-specific phosphorylation, or nuclear–cytoplasmic trafficking. Collectively, the data point to a functional connection among TCN1, STAT4, and the DUOX2/ROS axis that, by modulating oxidative stress, may constitute a tumor cell–autonomous oncogenic route in PDAC.

ROS exhibit concentration-dependent effects in tumor cells. Moderately elevated ROS levels promote tumor cell proliferation by activating the PI3K/AKT and MAPK signaling pathways [39]. In addition, ROS stabilize HIF-1α, thereby upregulating VEGF expression to facilitate angiogenesis. In the present study, DUOX2 functioned as an important source of ROS and was closely associated with EMT initiation and enhanced motility. TCN1 overexpression induced a moderate increase in ROS, consistent with the proliferative, invasive, and metastatic phenotypes of PDAC cells. These observations suggest that tumors may maintain a malignant advantage by tuning ROS homeostasis. Furthermore, the antioxidant N-acetylcysteine (NAC) and 4-Hydroxy-TEMPO (Tempol) effectively reversed TCN1-driven tumor progression, further supporting ROS as the critical effector molecule within this signaling axis.

From a biological process perspective, DUOX2-mediated EMT activation through ROS represents a core mechanism underlying PDAC metastasis. During EMT, the loss of E-cadherin and upregulation of N-cadherin/vimentin are critical hallmarks of acquired tumor invasiveness [40,41]. This study showed that TCN1 regulates EMT-related protein expression through the STAT4/DUOX2 axis, which is consistent with the differential outcomes observed in the hepatic metastasis model in vivo. Notably, this process is tightly linked to ROS generation. ROS promote EMT by activating the TGF-β and Wnt signaling pathways [42,43], and DUOX2, as a primary ROS source, likely drives metastasis in PDAC through analogous mechanisms. Furthermore, ROS-induced oxidative DNA damage exacerbates genomic instability, which may underlie the poor prognosis observed in patients with high TCN1 expression. Integrating transcriptional evidence with phenotypic assays, we propose that the TCN1/STAT4/DUOX2/ROS axis is an important driver of PDAC malignant progression; this model not only informs the underlying biology but also provides tractable entry points for therapeutic intervention.

It should be emphasized that this study has certain limitations. Current evidence supports a TCN1–STAT4 interaction and its impact on DUOX2/ROS and associated phenotypes, with regulation localized to post-transcriptional/ post-translational layers, but pathway- and site-level resolution is still lacking; how TCN1 influences STAT4 activity through protein stability and site-specific phosphorylation requires further investigation. In addition, although N-acetylcysteine (NAC) and the ROS scavenger Tempol were used to verify ROS as a key effector, both are pleiotropic pharmacological tools, and thus the specificity of pharmacologic interventions remains limited.

## 5. Conclusions

In summary, this study delineates a molecular framework whereby TCN1 promotes malignant progression of PDAC through a STAT4-mediated DUOX2/ROS signaling axis, implicating this pathway in proliferation, invasion, metastasis, and EMT and aligning with the adverse clinical outcomes associated with high TCN1 expression. Mechanistically, current evidence supports an interaction between TCN1 and STAT4 and a facilitatory effect on the DUOX2/ROS axis, but it remains insufficient to define the precise regulatory mechanism by which TCN1 modulates STAT4. Future investigations focusing on protein stability and post-translational modifications may identify actionable nodes, thereby providing a stronger basis for molecular subtyping, prognostic assessment, and precision therapeutic strategies in PDAC.

## Figures and Tables

**Table 1:** Correlation of TCN1 Expression Levels with Clinicopathological Characteristics.

Clinical Characteristic	Total	TCN1 Expression		*p* Value
		Low (40)	High (40)	
Age (years)				
<60	32	14	18	0.361
≥60	48	26	22	
Gender				
Male	44	23	21	0.653
Female	36	17	19	
TNM stage				
Ⅰ+Ⅱa	31	21	10	0.012 *
Ⅱb+Ⅲ	49	19	30	
Nodal metastasis				
Yes	45	20	25	0.259
No	35	20	15	
pathologic stage				
G1+G2	48	29	19	0.022 *
G3	32	11	21	

Clinicopathological characteristics of the PDAC cohort and their association with TCN1 expression. Data are presented as number of cases (n) in the low- and high-TCN1 groups (n = 40 each). *p* values were calculated using the X^2^ test (or Fisher’s exact test when appropriate). Statistically significant differences are marked with * (* *p* < 0.05). Staging followed AJCC 8th edition; grading followed WHO criteria.

## Data Availability

All data generated or analyzed in this study are included in the published article and its supplementary information files, and the TCGA and GEO datasets used are publicly available.

## Supplementary Materials

The following supporting information can be downloaded at: https://www.mdpi.com/article/10.3390/cancers17203300/s1, 1. Doc S1 (Additional material and methods). 2. Doc S2 (Supplementary figure legend). 3. Supplementary Figures S1–S11, Figure S1: Lentiviral modulation of TCN1 expression. Figure S2: TCN1 modulates EMT markers in vitro and in vivo. Figure S3: TCN1 regulates DUOX2 expression in vitro and in vivo. Figure S4: DUOX2 promotes proliferation in pancreatic cancer cells. Figure S5: DUOX2 enhances invasion, metastasis, and epithelial–mesenchymal transition (EMT) in pancreatic cancer. Figure S6: TCN1 regulates pancreatic cancer cell proliferation through DUOX2. Figure S7: TCN1 modulates invasion and metastasis through DUOX2. Figure S8: Impact of TCN1 modulation on STAT4 transcription and phosphorylation in PDAC cells. Figure S9: TCN1 regulates proliferation through STAT4. Figure S10: TCN1 alters invasive/metastatic behavior through STAT4. Figure S11: TCN1 drives malignant phenotypes through ROS.

## Abbreviations

PDAC: pancreatic ductal adenocarcinoma; TCN1: transcobalamin 1; STAT4: signal transducer and activator of transcription 4; DUOX2: dual Oxidase 2; ROS: reactive oxygen species; qRT-PCR: real-time quantitative reverse transcription-polymerase chain reaction; siRNAs: small interfering RNAs; si-NC: siRNA-negative control; shRNA: short hairpin RNA; shCtrl: shRNA control; EdU assay: ethynyl-2-deoxyuridine incorporation assay; WT: wild-type; MUT: mutated; IF: immunofluorescence; Co-IP: co-immunopreciptation; IHC: immunohistochemical; TCGA: The Cancer Genome Atlas; EMT: epithelial–mesenchymal transition; GTEx: genotype-tissue expression.
