# Supplementary material for: TCN1 Drives Malignant Progression of Pancreatic Cancer Through STAT4-Mediated Transcriptional Activation of the DUOX2/ROS Signaling Axis
Source: Cancers (Basel). 2025 Oct 12;17(20):3300. doi: 10.3390/cancers17203300 (PMC12563811; doi:10.3390/cancers17203300)
Supplement: Supplementary file 1 [file cancers-17-03300-s001.zip › cancers-3867328-original-images.pdf]

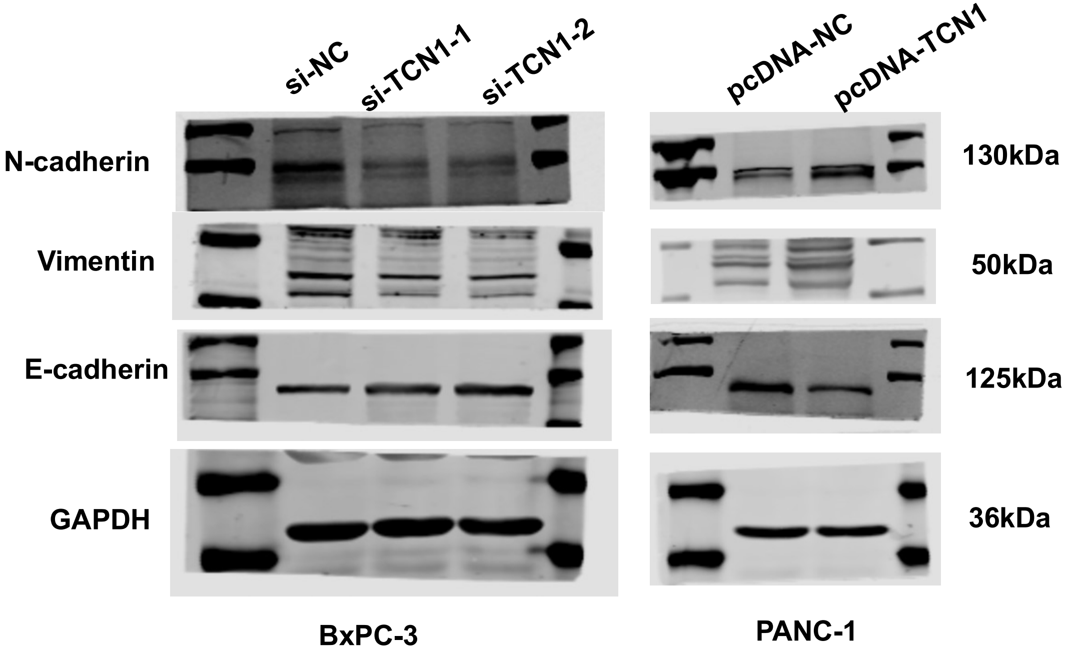

Original WB image for Figure 3I

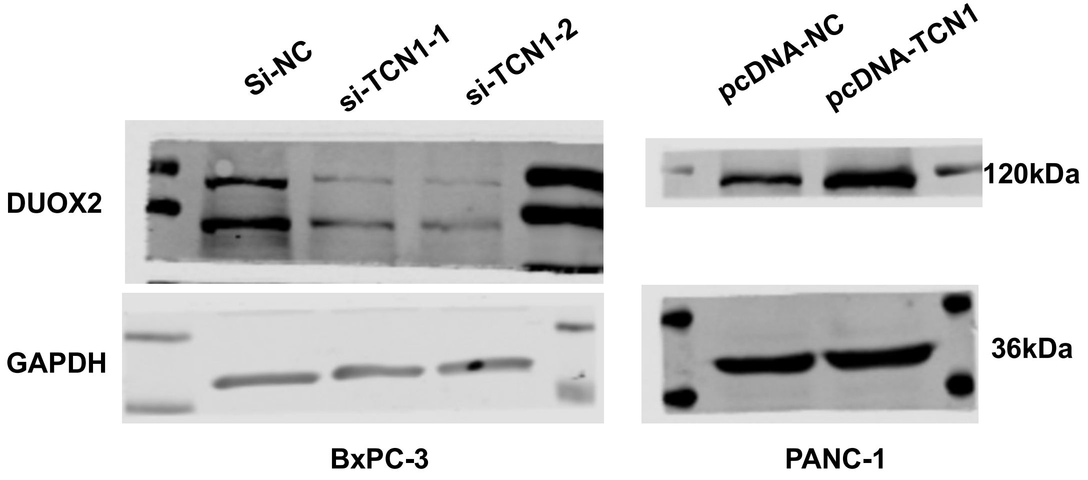

Original WB image for Figure 4D and E

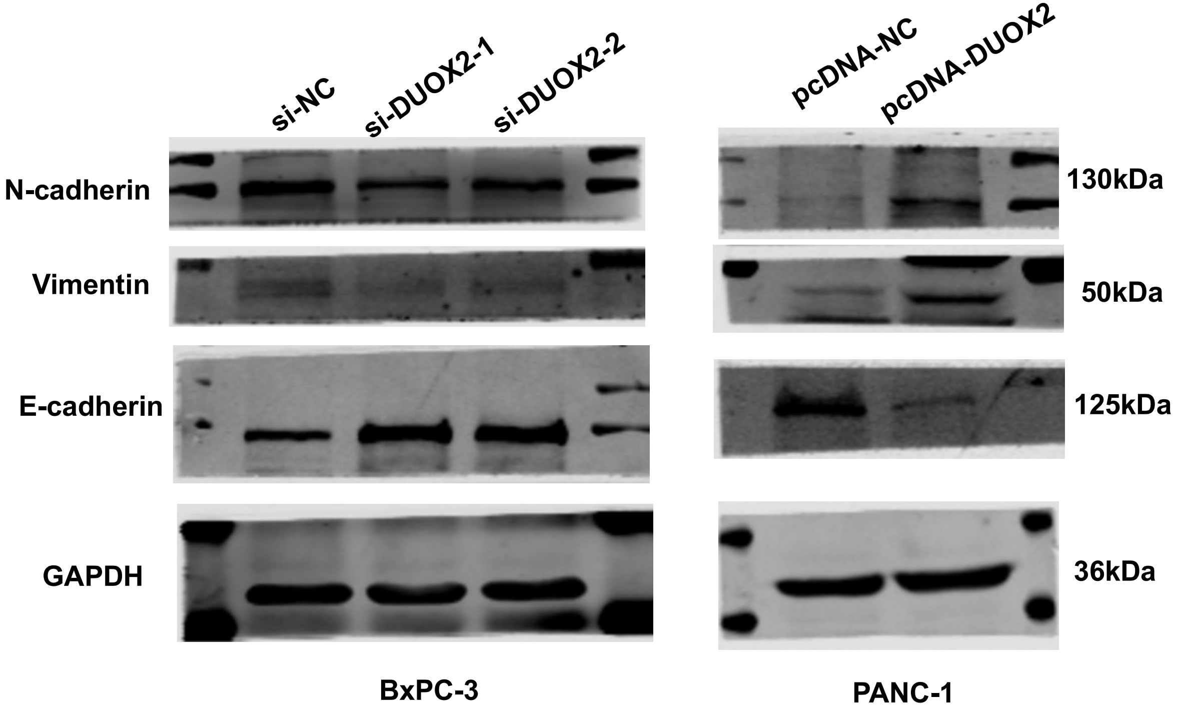

Original WB image for Supplementary FigureS5G

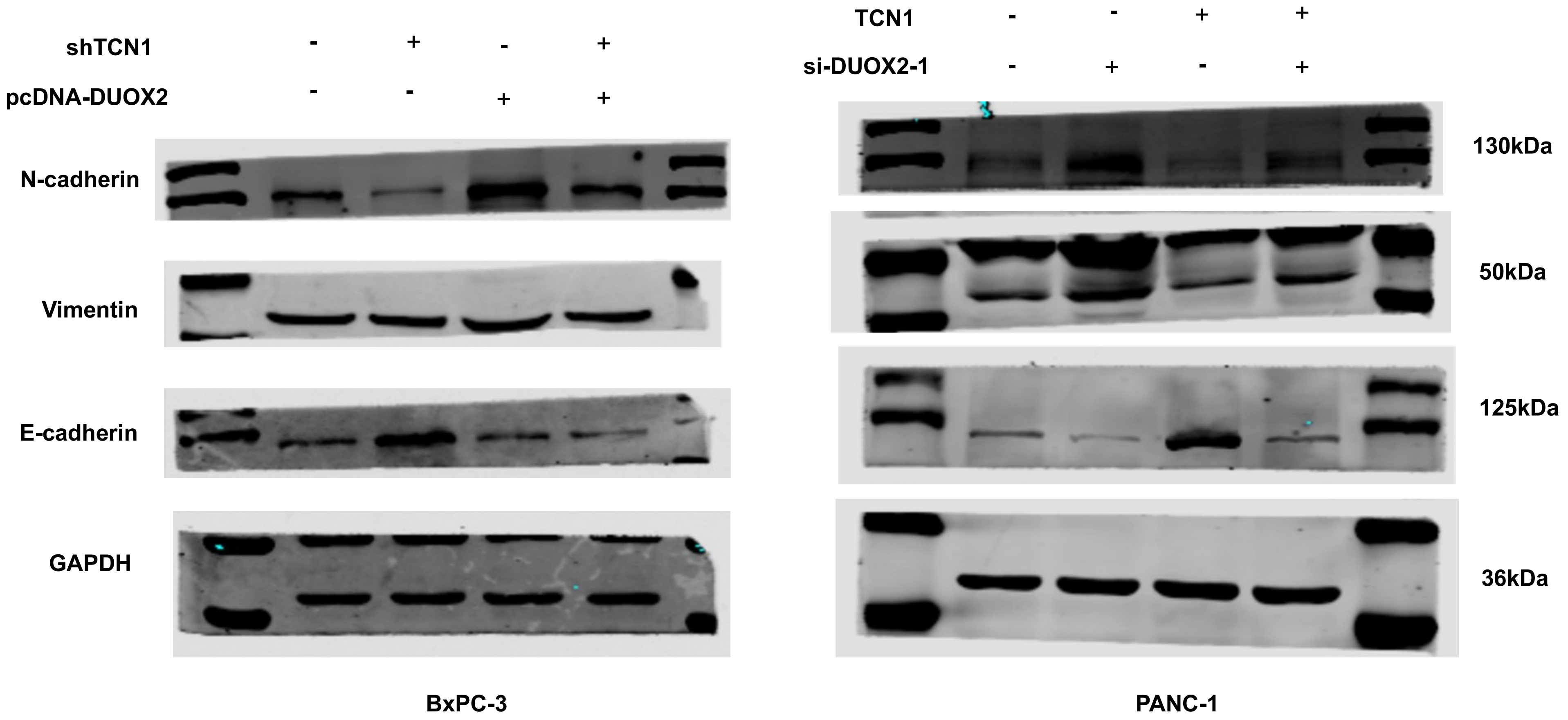

Original WB image for Figures 5G and H

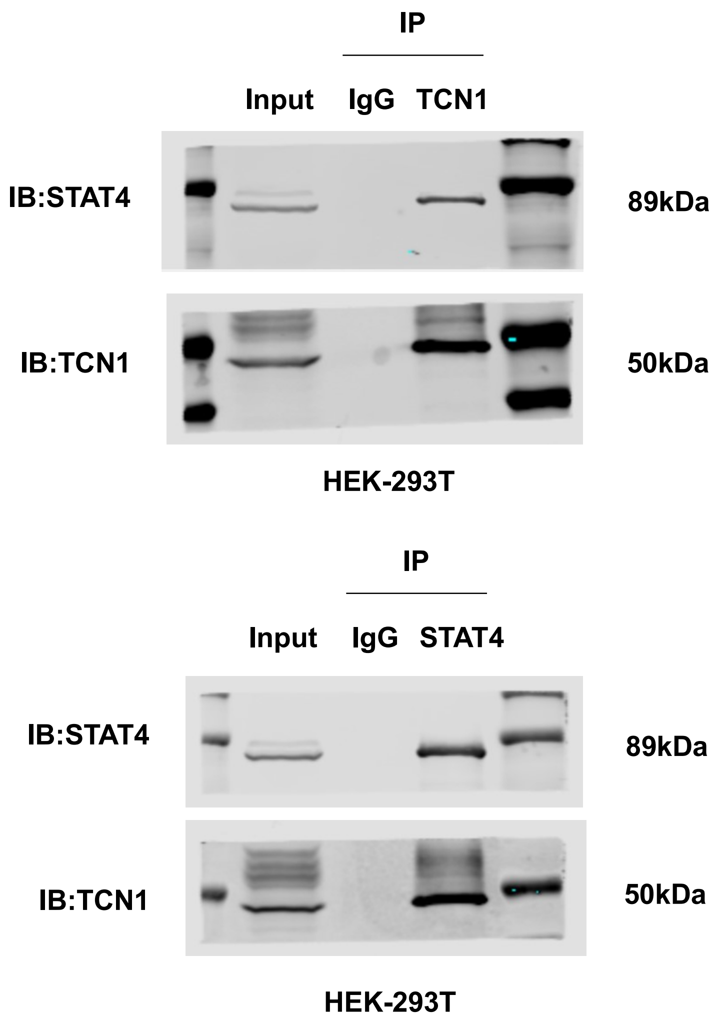

Original WB image for Figure 6C

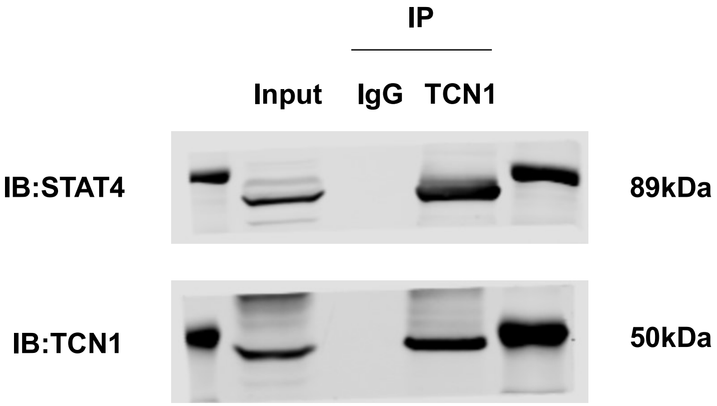

PANC-1

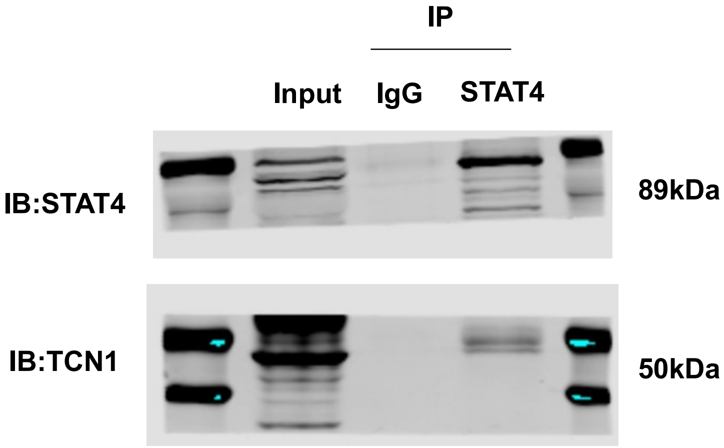

PANC-1

Original WB image for Figure 6D

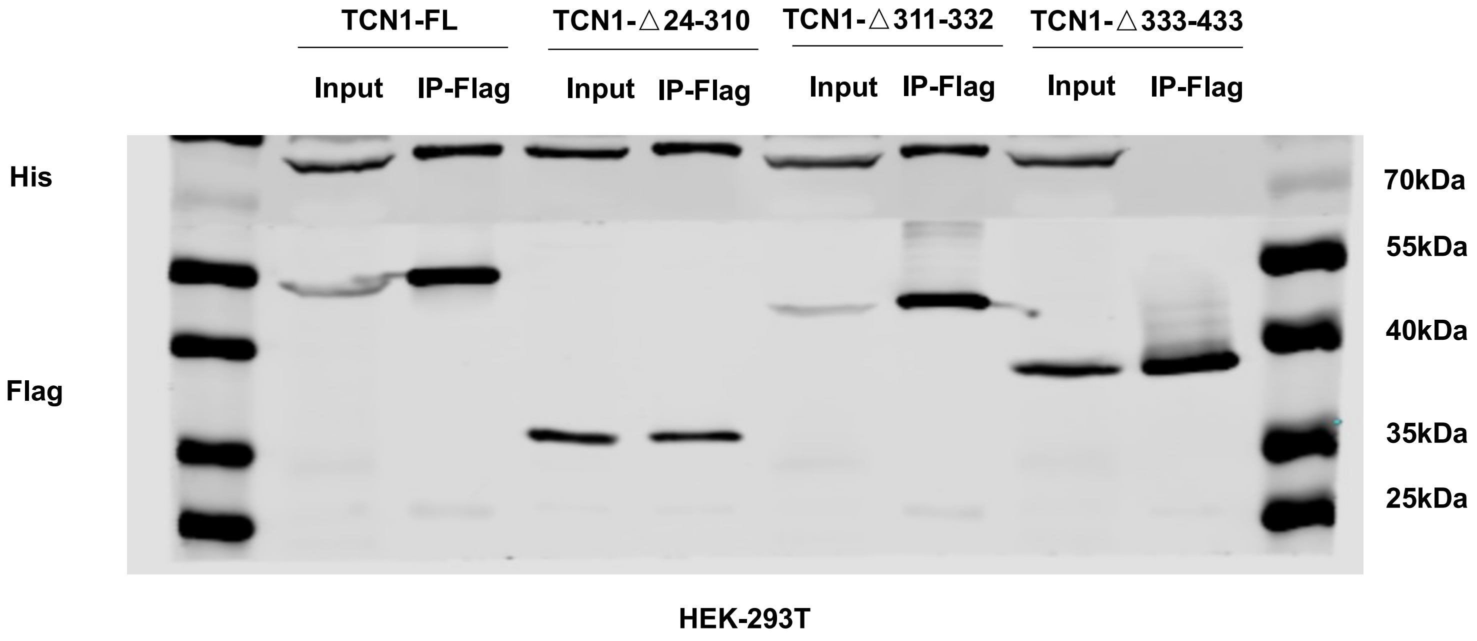

Original WB image for Figure 6H

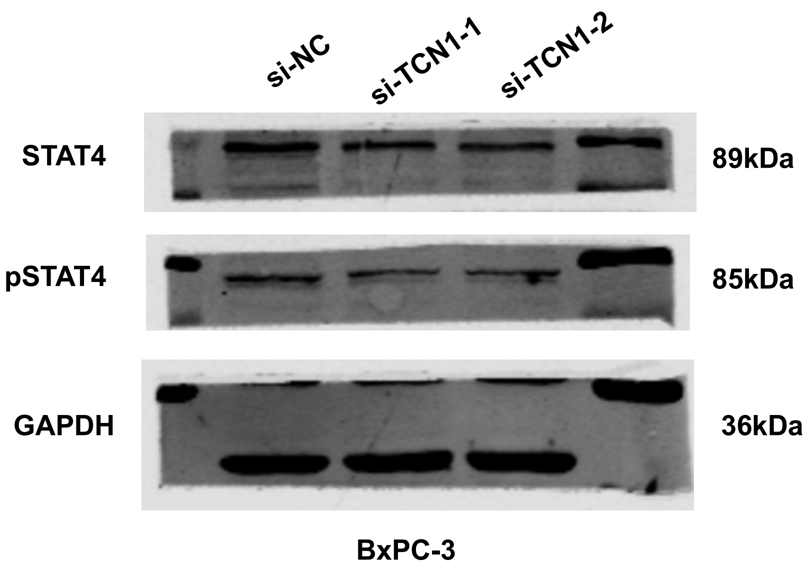

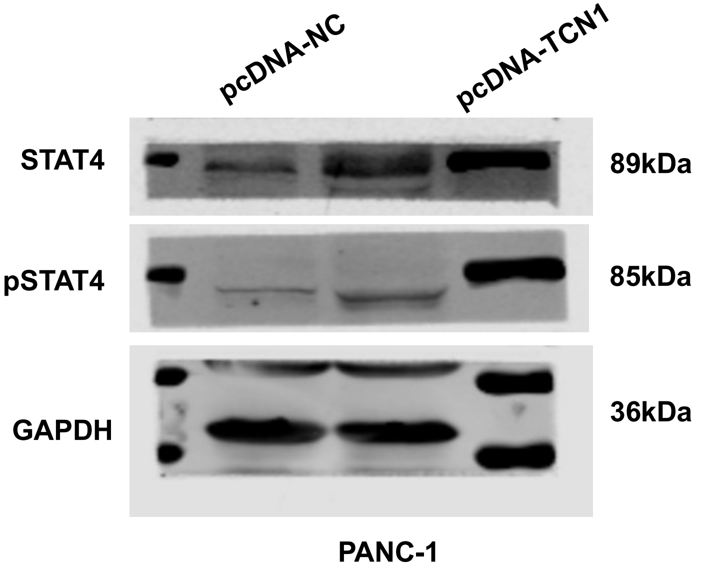

Original WB image for Figures 6I and J

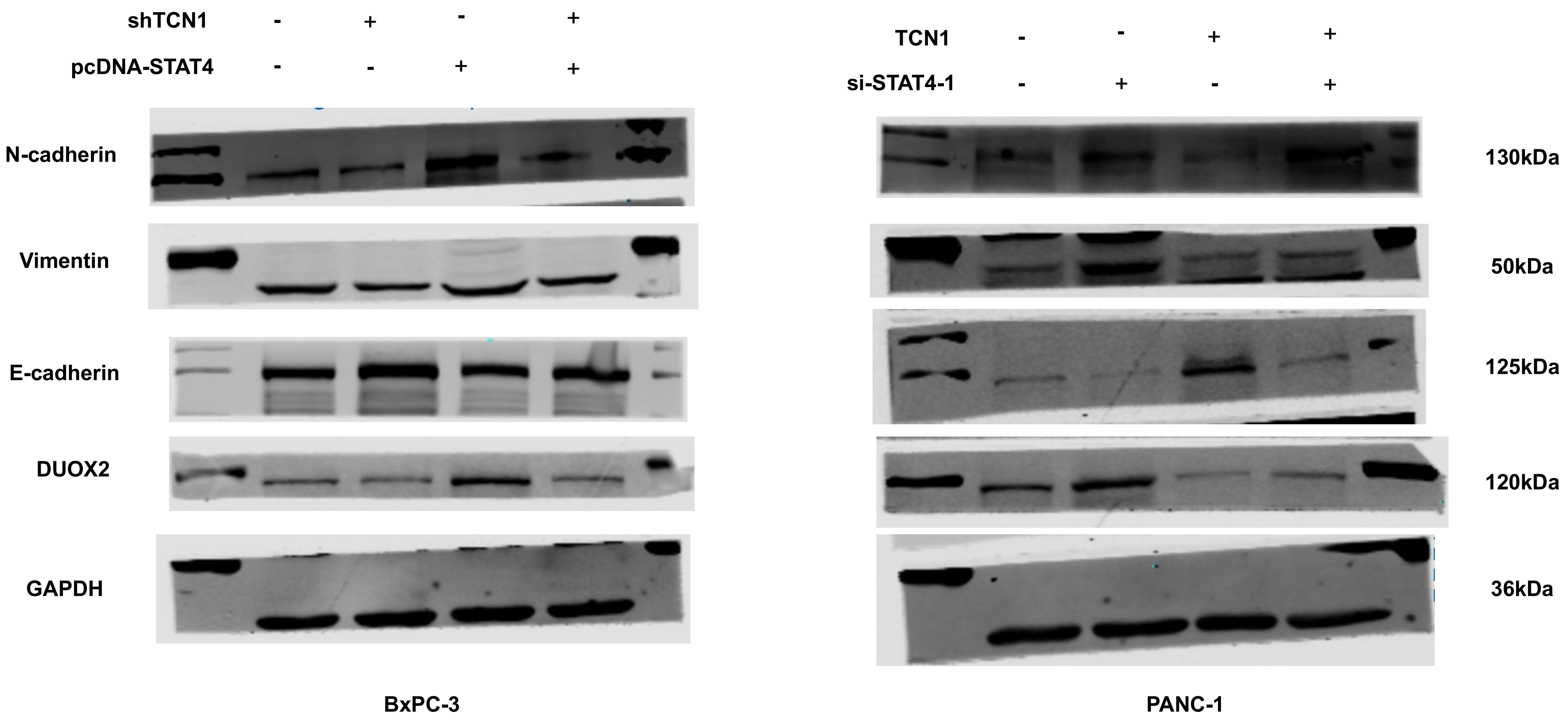

Original WB image for Figures 7E and F
