# Supplementary material for: TCN1 Drives Malignant Progression of Pancreatic Cancer Through STAT4-Mediated Transcriptional Activation of the DUOX2/ROS Signaling Axis
Source: Cancers (Basel). 2025 Oct 12;17(20):3300. doi: 10.3390/cancers17203300 (PMC12563811; doi:10.3390/cancers17203300)
Supplement: Supplementary file 1 [file cancers-17-03300-s001.zip › Doc S1.pdf]

Doc S1

1. In-silico analyses

In-silico analyses were performed using publicly available datasets and TF-binding site resources. Pancreatic ductal adenocarcinoma (tumor) data were obtained from TCGA-PAAD and normal pancreas data from GTEx. We compared TCN1 and DUOX2 expression between tumor and normal tissues and evaluated their associations with overall survival using Kaplan – Meier analysis with log-rank testing. Differential expression thresholds were preset at $|\log2FC| \geqslant 2$ with two-tailed $P < 0.05$. Gene–gene associations were examined and visualized in GEPIA. To define the upstream transcriptional mechanism, JASPAR and hTFtarget predictions were intersected to identify STAT4 motifs within the DUOX2 promoter; these candidate sites guided subsequent ChIP-qPCR and dual-luciferase validation experiments. Unless otherwise stated, statistical analyses were performed in SPSS 25.0 and GraphPad Prism 10.0, applying Pearson correlation and log-rank tests as appropriate, with significance defined as two-tailed $P < 0.05$.

2. Cell lines and culture conditions

Human pancreatic ductal adenocarcinoma cell lines (ASPC-1, BxPC-3, CFPAC-1, PANC-1, SW1990, and MIAPaCa-2), normal pancreatic ductal epithelial (HPDE) cells, and HEK-293T renal epithelial cells were obtained from the Cell Bank of Type Culture Collection of Chinese Academy of Sciences (Shanghai, China). ASPC-1 and BxPC-3 cells were maintained in RPMI-1640 medium (Gibco, USA), whereas PANC-1,

SW1990, MIAPaCa-2, and HEK-293T cells were cultured in Dulbecco's Modified Eagle Medium (DMEM; Gibco). CFPAC-1 cells required Iscove's Modified Dulbecco's Medium (IMDM; Gibco) for optimal growth. All media were supplemented with 10% fetal bovine serum (FBS; Gibco), 100 U/mL penicillin, and 100 μg/mL streptomycin (Sigma-Aldrich, USA), and cultures were incubated at 37 °C in a humidified, 5% CO_2 atmosphere. Cells were authenticated by STR profiling and confirmed mycoplasma-free by PCR monthly.

3. Cell transfection and viral infection

siRNAs targeting TCN1, STAT4, and DUOX2, as well as a non-targeting control (si-NC), were purchased from RiboBio (Guangzhou, China). For ectopic expression, human TCN1/STAT4/DUOX2 cDNAs were cloned into mammalian expression plasmids (Genechem, Shanghai, China). For transient experiments, cells were seeded in 6-well plates to 60–70% confluence in antibiotic-free medium and transfected in Opti-MEM using Lipofectamine 3000 (Thermo Fisher Scientific, USA) at 50 nM siRNA or 2 μg plasmid DNA per well; the medium was replaced after 6 h, and samples were collected 24–72 h post-transfection (typically ~48 h for knockdown and 48–72 h for overexpression). For stable manipulation, GV112 lentiviral vectors (HANHENG, Shanghai) carrying sh-TCN1, sh-DUOX2, sh-Ctrl, and TCN1/DUOX2 overexpression/empty constructs were used, and stable pools were obtained by puromycin selection (1–2 μg/mL) for 7–10 days. Knockdown/overexpression efficiency was confirmed by qRT-PCR and Western blot.

4. RNA extraction and quantitative reverse transcription-polymerase chain reaction (qRT-PCR) Analysis

Total RNA was extracted from snap-frozen tissues and cultured cells using the AxyPrep Multisystem Total RNA Miniprep Kit (Corning, Suzhou, China) with on-column DNase I treatment. RNA quantity/purity and integrity were assessed by spectrophotometry and agarose gel electrophoresis. First-strand cDNA was synthesized from 500–1,000 ng RNA in a 20 μL reaction using the ReverTra Ace qPCR RT Kit (FSQ-301; Toyobo, Osaka, Japan). qRT-PCR was performed on a 7500 FAST Real-Time PCR System (Applied Biosystems, USA) with a SYBR Green 2× master mix (20 μL reactions; primer 0.2 μM). Cycling was 95 °C 30 s, then 40 cycles of 95 °C 5–10 s and 60 °C 30 s, followed by a melt curve to confirm specificity. Primer sequences (amplicons 80–200 bp) are listed in Supplementary Table S1. Each sample was run in technical triplicate with ≥3 biological replicates. GAPDH served as the internal control, and relative expression was calculated by the $2^{-\Delta\Delta Ct}$ method using the indicated control group as calibrator. No-template and no-RT controls were included.

5. Western blot analysis

Total protein was extracted from PDAC tissues and cultured cells using RIPA buffer (Beyotime, Beijing, China) supplemented with protease and phosphatase inhibitors. Tissues were homogenized on ice; cells were lysed on ice for 20–30 min and clarified by centrifugation (12,000 ×g, 10 min, 4 °C). Protein concentration was measured by BCA. Samples were mixed with Laemmli buffer containing a reducing agent and

denatured (95 °C, 5 min). Equal amounts (30 µg) were separated by SDS-PAGE (10% gels) and transferred to 0.45 µm nitrocellulose membranes by wet transfer. Membranes were blocked in 5% milk/TBST (for phospho-proteins, 5% BSA), incubated with primary antibodies overnight at 4 °C, then with near-infrared secondary antibodies (LI-COR) for 1 h at room temperature. After TBST washes, signals were acquired on an Odyssey CLx (700/800 nm). Bands were quantified in Image Studio/ImageJ and normalized to GAPDH or β-actin (phospho-proteins additionally to total protein). Immunoblots were repeated in ≥3 biological replicates. Antibody sources and dilutions are provided in Supplementary Table S2.

6. Co-immunoprecipitation (Co-IP)

Cells were lysed on ice for 10 min in Pierce™ IP Lysis Buffer (Thermo Fisher Scientific, Waltham, MA, USA) supplemented with 1× protease and 1× phosphatase inhibitors. For each IP, 500 µL lysate from 1.0×107 cells was prepared, clarified at 12,000 × g for 10 min at 4 °C, adjusted to 2.0 mg/mL (BCA), and 25 µL was reserved as input. BeyoMag™ Protein A+G beads were equilibrated by washing with 500 µL lysis buffer 2 times (2 min each on a magnetic rack). Pre-clearing was performed by adding 20 µL equilibrated Protein A+G beads to 500 µL lysate and rotating 60 min at 22 °C (20 rpm); beads were captured 2 min on a magnet. The supernatant was then incubated with 30 µL Mouse IgG beads (negative control) or 30 µL Anti-Flag/Anti-His beads (Beyotime Institute of Biotechnology, Beijing, China) for 120 min at 22 °C (20 rpm). Bead–protein complexes were washed 3 times with 1.0 mL 1× TBS (50 mM Tris-HCl, 150 mM NaCl, pH 7.4), 5 min each at 22 °C, followed by magnetic separation (2

min) after each wash. Proteins were eluted in 40 μL 2× Laemmli buffer containing 5% β-mercaptoethanol at 95 °C for 5 min. For Western blot, 10 μL eluate and 10 μL input (boiled with 10 μL 2× Laemmli) were loaded per lane.

7. Immunofluorescence (IF)

All colocalization results in this study were obtained by immunodetection of endogenous TCN1 and endogenous STAT4 at native expression levels; no epitope tags, fluorescent fusions, or overexpression constructs were used.PANC-1 and HEK-293T cells were cultured on #1.5 glass coverslips placed in 24-well plates. Cells were fixed with 4% paraformaldehyde in PBS for 15 min at 22 °C, then washed 3 times with PBS containing 0.1% Triton X-100, 5 min each, 500 μL/well. Non-specific binding was blocked with 5% BSA in PBS, 300 μL/well, for 30 min at 22 °C. Primary antibodies were applied in 1% BSA/PBS at the dilutions specified in Supplementary Table S2, 200 μL/well, for 120 min at 22 °C. After 3 washes with PBS, 5 min each, 500 μL/well, species-appropriate fluorescent secondary antibodies (see Supplementary Table S2 for catalog and dilution) were added in 1% BSA/PBS, 200 μL/well, for 60 min at 22 °C in the dark. Nuclei were counterstained with DAPI (1 μg/mL in PBS), 200 μL/well, for 5 min at 22 °C in the dark, followed by 2 final PBS rinses (500 μL/well). Coverslips were mounted with anti-fade mounting medium (Beyotime, Beijing, China) and imaged on an Eclipse Ti2 inverted fluorescence microscope (Nikon, Tokyo, Japan). Identical exposure and gain settings were applied across groups. Antibody specifications (source, clonality and dilution) are listed in Supplementary Table S2.

8. Chromatin Immunoprecipitation (ChIP)

Chromatin immunoprecipitation was performed with the BeyoChIP™ Enzymatic ChIP Assay Kit (Beyotime Institute of Biotechnology, Beijing, China). For each IP, 1.0×107 cells in 10-cm dishes were cross-linked to a final 37% formaldehyde for 10 min at 22 °C and quenched with 125 mM glycine for 5 min at 22 °C. Cells were washed 2× with ice-cold PBS (10 mL each), pelleted (500 × g, 5 min, 4 °C), and lysed in 1,000 µL kit SDS lysis buffer on ice for 10 min. Chromatin was sheared with the kit Enzymatic Shearing Mix at 37 °C for 12 min (300 rpm) to 200-300 bp; the reaction was stopped with 10 µL 0.5 M EDTA (final 50 mM), diluted to 500 µL, and 25 µL was reserved as input.For IP, 475 µL chromatin was incubated with 10 µg Phospho-Stat4 antibody (CST, Boston, USA) or 2 µg control IgG (CST) for 16 h at 4 °C (20 rpm). Protein A/G Magnetic Beads (30 µL; Beyotime) were pre-blocked in 500 µL ChIP dilution buffer containing 0.5 mg/mL BSA and 0.2 mg/mL salmon sperm DNA for 30 min at 22 °C, then added to the antibody–chromatin mixture for 60 min at 22 °C (20 rpm). Bead–chromatin complexes were washed sequentially with kit Low-Salt, High-Salt, LiCl, TE, and TE buffers (1.0 mL, 5 min at 22 °C, magnetic separation 2 min between washes). DNA was eluted in 100 µL kit Elution Buffer at 65 °C for 15 min (1,000 rpm), crosslinks were reversed by adding NaCl to 200 mM and incubating at 65 °C for 6 h, followed by RNase A 10 µg (37 °C, 30 min) and Proteinase K 20 µg (55 °C, 60 min). DNA was purified on the kit column and eluted in 50 µL nuclease-free water. 1 µL ChIP DNA was used per 20 µL SYBR qPCR.

9. Dual-luciferase reporter assay

Dual-luciferase assays used the Dual-Luciferase® Reporter Assay System (Promega). BxPC-3 and PANC-1 cells were seeded in white 96-well plates at 1.5×104 cells/well in 100 μL medium. Per well, 200 ng DNA in 10 μL Opti-MEM was transfected with Lipofectamine 3000: 100 ng DUOX2-promoter firefly reporter (WT or MUT), 10 ng Renilla control (pRL-TK), 90 ng expression or empty vector. After 6 h, replace with 100 μL fresh medium. At 48 h, lyse in-well with 40 μL 1× Passive Lysis Buffer for 15 min at 22 °C (plate shaker 500 rpm). Measure firefly by adding 20 μL LAR II (Varioskan Flash; 2 s delay, 10 s integration), then Renilla by adding 20 μL Stop & Glo® with the same settings. Compute RLU = Firefly/Renilla. Each condition used 3 technical wells and 3 biological replicates.

10. Silver staining

Silver staining was performed with the Fast Silver Stain Kit (Beyotime, Beijing, China). Protein (30 μg/lane) was resolved on 10% SDS-PAGE, 1.0-mm gels in Tris–glycine–SDS buffer (25 mM Tris, 192 mM glycine, 0.1% SDS) at 120 V, 22 °C, 80 min. Gels were fixed in 25 mL Fixing Solution for 40 min, washed with 25 mL 30% ethanol (10 min), 25 mL water (5 min), 25 mL 30% ethanol (10 min), and 25 mL water (5 min), sensitized in 25 mL 1× Sensitizing Solution (2 min), rinsed twice with 25 mL water (1 min each), impregnated in 25 mL 1× Silver Solution (10 min, 22 °C), rinsed twice with 25 mL water (30 s each), developed in 25 mL 1× Developing Solution (3 min, 22 °C), and stopped with 25 mL 1× Stop Solution (5 min). Images were acquired on a gel documentation system.

11. EDU and clonogenic assays

Cells were seeded in 24-well plates at 5.0×104 cells/well in 500 µL medium and incubated 24 h. EdU labeling used 10 µM working solution (500 µL/well, 120 min, 37 °C, 5% CO_2), followed by PBS washes 2× (500 µL each), fixation (4% PFA/PBS, 500 µL, 15 min, 22 °C), and permeabilization (0.3% Triton X-100/PBS, 500 µL, 15 min, 22 °C). The click reaction cocktail was applied (250 µL, 30 min, 22 °C, dark), nuclei were counterstained with Hoechst 33342 (1 µg/mL, 200 µL, 5 min, 22 °C, dark), and wells were rinsed PBS 2× (500 µL each). Images were acquired on a Nikon Eclipse Ts2R-FL microscope (20×); 5 fields/well were analyzed in ImageJ to calculate EdU^+ fraction = EdU^+ nuclei / Hoechst nuclei × 100%. Each condition used 3 technical wells and 3 biological replicates.

For clonogenic survival assays , exactly 1,000 cells/well were plated in 6-well plates with 2.0 mL/well medium and cultured 14 d at 37 °C, 5% CO_2, with medium changes on day 4 and day 8 (2.0 mL each). Colonies were fixed (4% PFA/PBS, 1.0 mL, 15 min, 22 °C), rinsed with water 2× (1.0 mL each), stained (0.1% crystal violet/20% methanol, 1.0 mL, 20 min, 22 °C), washed with water 3× (1.0 mL each), and air-dried 30 min. Plates were imaged and colonies (≥50 cells) counted in ImageJ. Each condition used triplicate wells and 3 biological replicates.

12. Transwell and wound healing assays

For Transwell invasion assays, 5×10^4 cells resuspended in 200 µL serum-free medium were seeded into Falcon® inserts (8 µm pore size; Corning, USA) pre-coated with or

without Matrigel® (BD Biosciences, USA). The lower chamber was filled with 500 μL complete medium. After 24 or 48 h of incubation, cells invading the lower membrane surface were fixed with 4% PFA and stained with 0.1% crystal violet for quantification. For each insert, 5 non-overlapping fields were counted in ImageJ; data are reported as cells/field. Each condition used n=3 inserts and 3 biological replicates.

For wound healing assays, transfected cells in 6-well plates were pretreated with mitomycin C 10 μg/mL (2.0 mL/well, 120 min, 37 °C), washed with PBS (2×, 2.0 mL each), and scratched with a 200 μL pipette tip along a ruler guide. Wells were filled with 2.0 mL serum-free medium (PANC-1) or 1% FBS medium (BxPC-3) and incubated 24 h at 37 °C, 5% CO_2. Phase-contrast images were captured at 0 h and 24 h (inverted microscope, 10×). Wound width was measured at 5 marked positions/well in ImageJ. Each condition used n=3 wells and 3 biological replicates.

13. Reactive oxygen species (ROS) detection

Intracellular ROS levels were quantified using the ROS Assay Kit (Beyotime Institute of Biotechnology, Beijing, China). Cells were loaded with 2',7'-dichlorodihydrofluorescein diacetate (DCFH-DA) at a 1:1 000 dilution for 20 min at 37 °C in the dark, followed by three washes with 1× PBS (pH 7.4). Fluorescence intensity was analyzed using an Eclipse Ti2 inverted fluorescence microscope (Nikon, Tokyo, Japan) with the NIS-Elements imaging software. Each condition used n=3 wells and 3 biological replicates.

14. Immunohistochemical (IHC) staining

FFPE tissues were sectioned at 4 μm, mounted, and baked at 60 °C for 60 min. Slides were deparaffinized in xylene for 10 min, twice, rehydrated sequentially in 100% ethanol for 5 min, twice, then 95% ethanol for 5 min, followed by 80% ethanol for 5 min, and finally rinsed in water for 5 min. Antigen retrieval was performed in 10 mM sodium citrate (pH 6.0) at 98 °C for 20 min, followed by cooling at room temperature for 20 min. Endogenous peroxidase was quenched with 3% H_2O_2 in methanol for 10 min at room temperature. After washing in PBS-T (0.05% Tween-20) for 5 min, three times, slides were blocked with 5% BSA in PBS for 30 min at room temperature.

Primary antibodies to TCN1, DUOX2, N-cadherin, vimentin, E-cadherin, and Ki-67 were applied at the dilutions listed in Supplementary Table S2 (prepared in 1% BSA/PBS) and incubated for 16 h at 4 °C. Following three 5-min washes in PBS-T, an HRP-conjugated secondary antibody (1:200 in PBS) was added for 30 min at room temperature. Slides were washed again in PBS-T for 5 min, three times, developed with DAB for 2 min, and the reaction was stopped in water for 2 min. Sections were counterstained with hematoxylin for 60 s, blued in 0.1% ammonia water for 30 s, dehydrated sequentially in 80% ethanol for 1 min, then 95% ethanol for 1 min, and 100% ethanol for 1 min, twice, cleared in xylene for 5 min, twice, and mounted. Images were captured on a Nikon Eclipse microscope at 200×; For quantification, five non-overlapping random fields per section were evaluated using the H-score method.

15. Orthotopic and hepatic metastasis models

All in vivo procedures were approved by the Animal Ethics Committee of the First

Affiliated Hospital of Harbin Medical University. Male BALB/c nude mice (5 weeks, ~20 g) were maintained under SPF conditions (22 °C, 50% humidity, 12 h light/12 h dark). Sample size: orthotopic pancreas model n=5; portal-vein hepatic metastasis model n=3. Lentivirus-infected cell lines were washed and resuspended in 50% Matrigel (Corning), with all procedures maintained on ice. Using a microsyringe (Hamilton), 2×10^6 cells in 10 μL suspension were injected into the vascularized pancreatic lobe. Weekly monitoring was performed using the Night OWLII LB 983 in vivo imaging system (Berthold Technologies GmbH & Co. KG). For hepatic metastasis induction, virus-infected cells were resuspended in 1× PBS, and 100 μL of cell suspension containing 5×10^6 cells was injected into the portal vein using a microsyringe. Following hemostasis and surgical closure of the abdominal incision, mice were euthanized at 8 weeks post-implantation for endpoint analysis.

16. Statistical analysis

Statistical analyses were performed in SPSS 25.0 (IBM) and GraphPad Prism 10.0 (GraphPad Software). Continuous variables are presented as mean ± SD. Unless otherwise stated, each experiment was independently repeated three times (biological replicates); for plate-based assays, three technical replicates per experiment were included. For two-group comparisons, two-tailed Student's t-tests were used; for ≥3 groups, one-way ANOVA with Tukey's post hoc test.Correlations were assessed with Pearson's coefficient (two-tailed). Kaplan–Meier survival curves were generated and compared by the log-rank test. Statistical significance was defined as two-tailed $P < 0.05$. The figure legends specify n (biological replicate or animal) for each analysis.

Supplementary table1. Primer sequences for PCR

Genes		Sequences
TCN1	TCN1-F	CAGTGTGATGGAGAAAGCCCAG
	TCN1-R	CCACTCAGAAGTTCCCAGTAGG
NUPR1	NUPR1-F	GACTCCAGCCTGGATGAATCTG
	NUPR1-R	CTTCTCTCTTGGTGCGACCTTTC
MMP1	MMP1-F	ATGAAGCAGCCCAGATGTGGAG
	MMP1-R	TGGTCCACATCTGCTCTTGGCA
C1R	C1R-F	TGGAGGCTACTTCTGTTCCTGC
	C1R-R	TACTCCAGGCTGGAGATGTAGC
IFI44L	IFI44L-F	TGCACTGAGGCAGATGCTGCG
	IFI44L-R	TCATTGCGGCACACCAGTACAG
CYP4B1	CYP4B1-F	CTGGACTTCCTGGACATTCTCC
	CYP4B1-R	GCCATGCAGTAGAGAAACCAGG
DUOX2	DUOX2-F	CAATGGCTACCTGTCCTTCCGA
	DUOX2-R	GTCCTTGGAGAGGAAGCCATTC
AKR1C2	AKR1C2-F	CCGAAGCAAGATTGCAGATGGC
	AKR1C2-R	TTTCAGTGACCTTTCCAAGGCTG
H1F0	H1F0-F	GTGAGAACGCTGACTCGCAGAT
	H1F0-R	GCCACTGACTTCTTGGGTTCGT
HLA-B	HLA-B-F	CTGCTGTGATGTGTAGGAGGAAG
	HLA-B-R	GCTGTGAGAGACACATCAGAGC

STAT4	STAT4-F	CAGTGAAAGCCATCTCGGAGGA
	STAT4-R	TGTAGTCTCGCAGGATGTCAGC
CALB1	CALB1-F	TTTCCTGCTGCTCTTCCGATGC
	CALB1-R	GCTCCTCAGTTTCTATGAAGCCA
GAPDH	GAPDH-F	CCTCTTGACTTCAACAGCGACCAC
	GAPDH-R	TGGTCCAGGGGTCTTACTCC

Supplementary table2. Primary antibodies for WB, IHC, and IF

Antibody	Concentration for WB	Concentration for IHC	Concentration for IF	Specificity	Company
N-cadherin	1:2 000	1:200		Rabbit polyclonal	Proteintech
Vimentin	1:4 000	1:2 000		Rabbit polyclonal	Proteintech
E-cadherin	1:2 000	1:400		Rabbit polyclonal	Proteintech
Ki-67		1:200		Rabbit polyclonal	Proteintech
TCN1	1:1 000	1:200	1:400	Rabbit polyclonal	Abcam
DUOX2	1:1 000	1:200		Rabbit polyclonal	ORIGENE
STAT4	1:2 000		1:500	Mouse monoclonal	Proteintech
pSTAT4	1:1 000			Rabbit monoclonal	CST
DYKDDDDK tag	1:5 000			Rabbit ponoclonal	Proteintech
His Tag	1:5 000			Mouse monoclonal	Proteintech
GAPDH	1:50 000			Mouse monoclonal	Proteintech
