# Supplementary material for: TCN1 Drives Malignant Progression of Pancreatic Cancer Through STAT4-Mediated Transcriptional Activation of the DUOX2/ROS Signaling Axis
Source: Cancers (Basel). 2025 Oct 12;17(20):3300. doi: 10.3390/cancers17203300 (PMC12563811; doi:10.3390/cancers17203300)
Supplement: Supplementary file 1 [file cancers-17-03300-s001.zip › Doc S2.pdf]

Supplementary Figure S1. Lentiviral modulation of TCN1 expression.

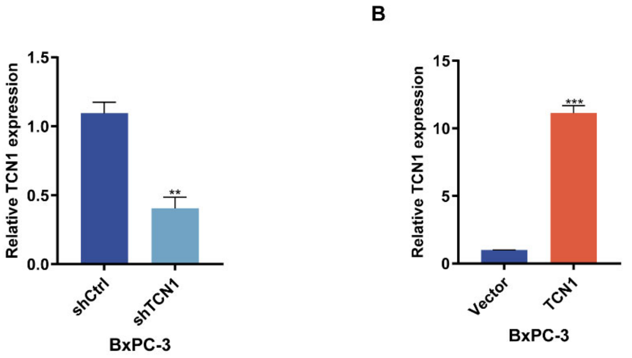

(A, B) qRT-PCR validation of TCN1 knockdown or overexpression efficiency following lentiviral transduction. Data are represented as means ± SD; *$P < 0.05$, **$P < 0.01$, ***$P < 0.001$.Three independent experiments were performed. Data is displayed as the mean ± SD. ns: no significance; *P < 0.05; **P < 0.01; ***P < 0.001.

Supplementary Figure S2. TCN1 modulates EMT markers in vitro and in vivo.

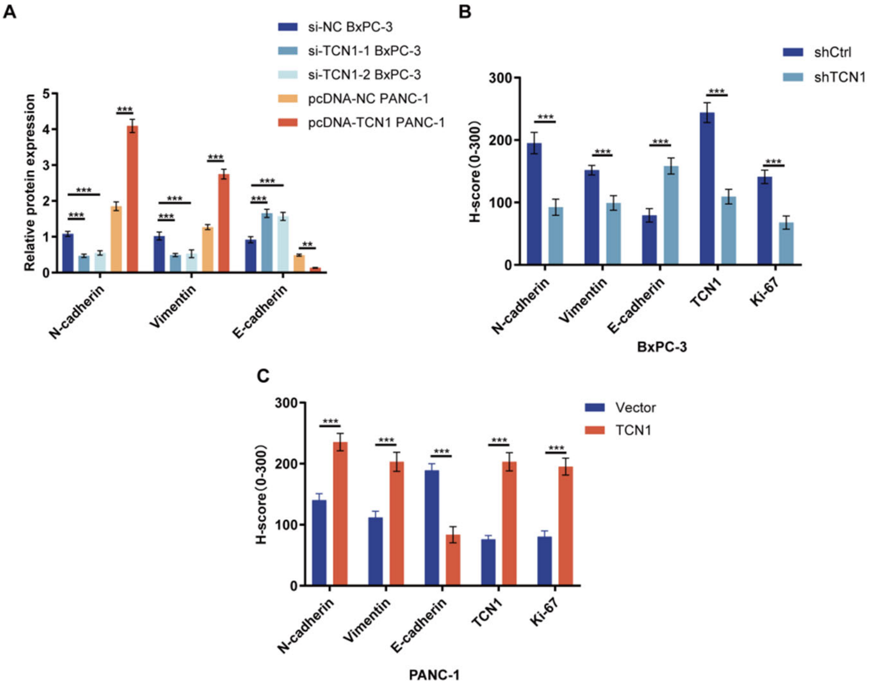

(A) Densitometric quantification of Western blots showing EMT markers (N-cadherin, vimentin, and E-cadherin) after TCN1 knockdown in BxPC-3 cells and overexpression in PANC-1 cells.(B, C) Immunohistochemical H-scores (0–300) for N-cadherin, vimentin, E-cadherin, TCN1, and Ki-67 in in-vivo tumors. Three independent experiments were performed. Data is displayed as the mean ± SD. ns: no significance; *P < 0.05; **P < 0.01; ***P < 0.001.

Supplementary Figure S3. TCN1 regulates DUOX2 expression in vitro and in vivo

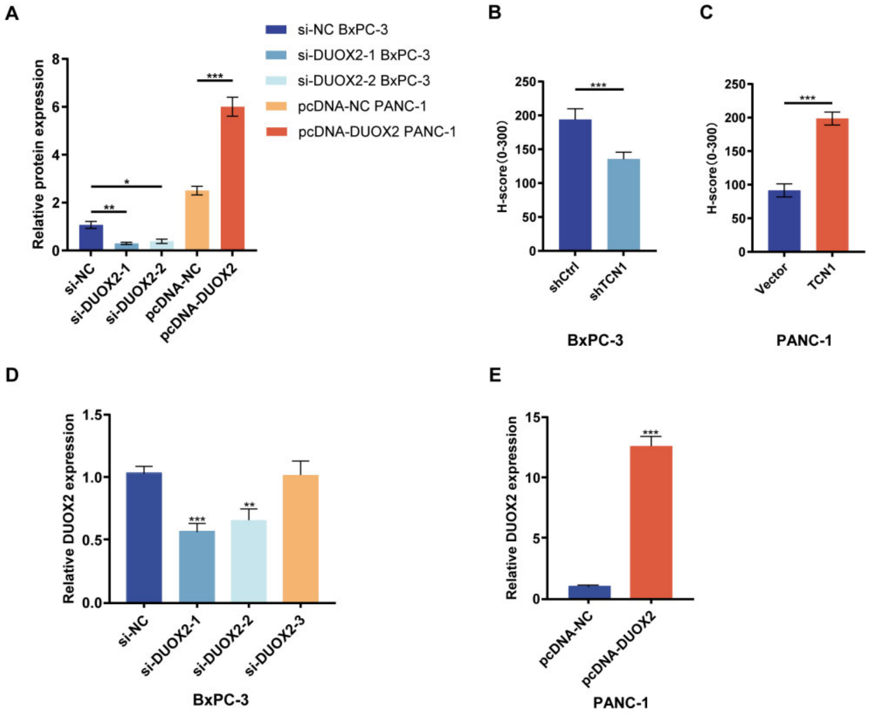

(A) Densitometric quantification of DUOX2 protein after TCN1 knockdown in BxPC-3 cells and overexpression in PANC-1 cells. (B, C) Immunohistochemical H-scores (0-300) of DUOX2 in in-vivo tumors derived from BxPC-3 cells with stable TCN1 silencing and from PANC-1 cells with TCN1 overexpression. (D, E)：

qRT-PCR validation of DUOX2 knockdown or overexpression efficiency in BxPC-3 and PANC-1 cells transfected with three distinct siRNAs or a pcDNA-DUOX2 plasmid.Three independent experiments were performed. Data is displayed as the mean ± SD. ns: no significance; *P < 0.05; **P < 0.01; ***P < 0.001.

Supplementary Figure S4. DUOX2 promotes proliferation in pancreatic cancer cells.

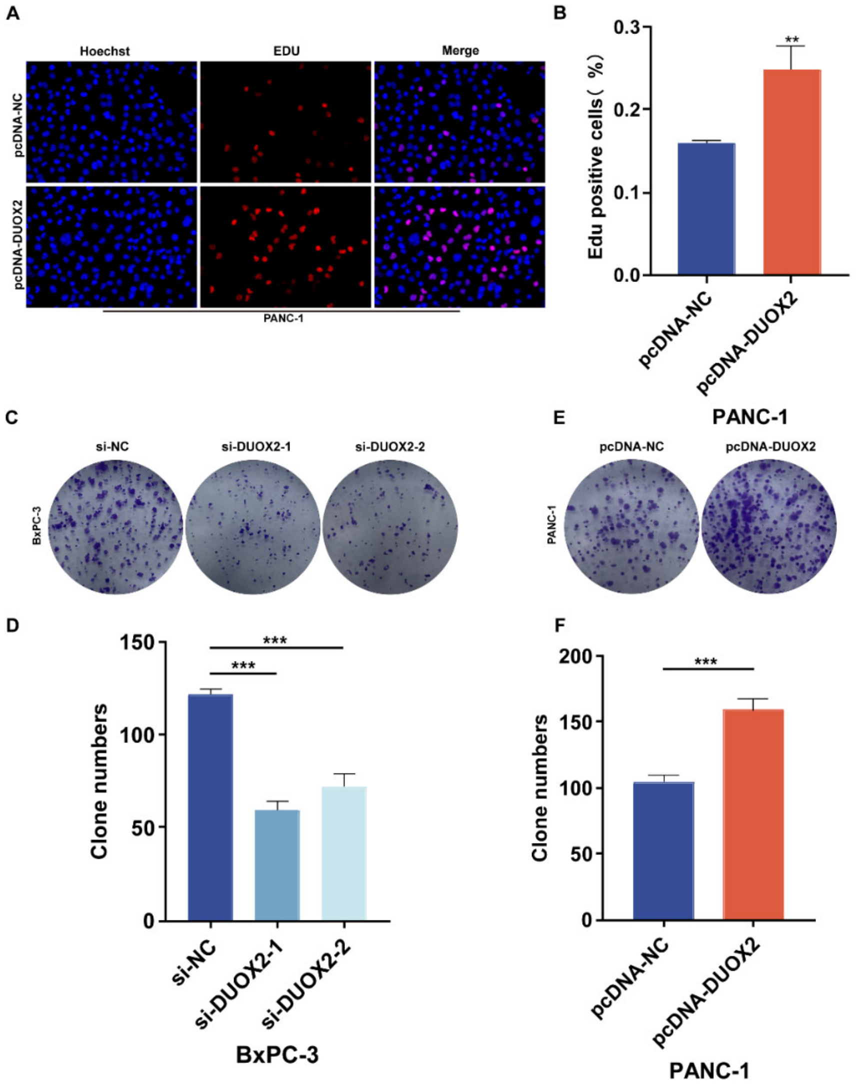

(A, B) EdU assay evaluating the proliferative effect of DUOX2 overexpression. (C–F)

Colony formation assays assessing the impact of DUOX2 knockdown or overexpression on cell proliferation. Three independent experiments were performed. Data is displayed as the mean ± SD. ns: no significance; *P < 0.05; **P < 0.01; ***P < 0.001.

Supplementary Figure S5. DUOX2 enhances invasion, metastasis, and epithelial–mesenchymal transition (EMT) in pancreatic cancer.

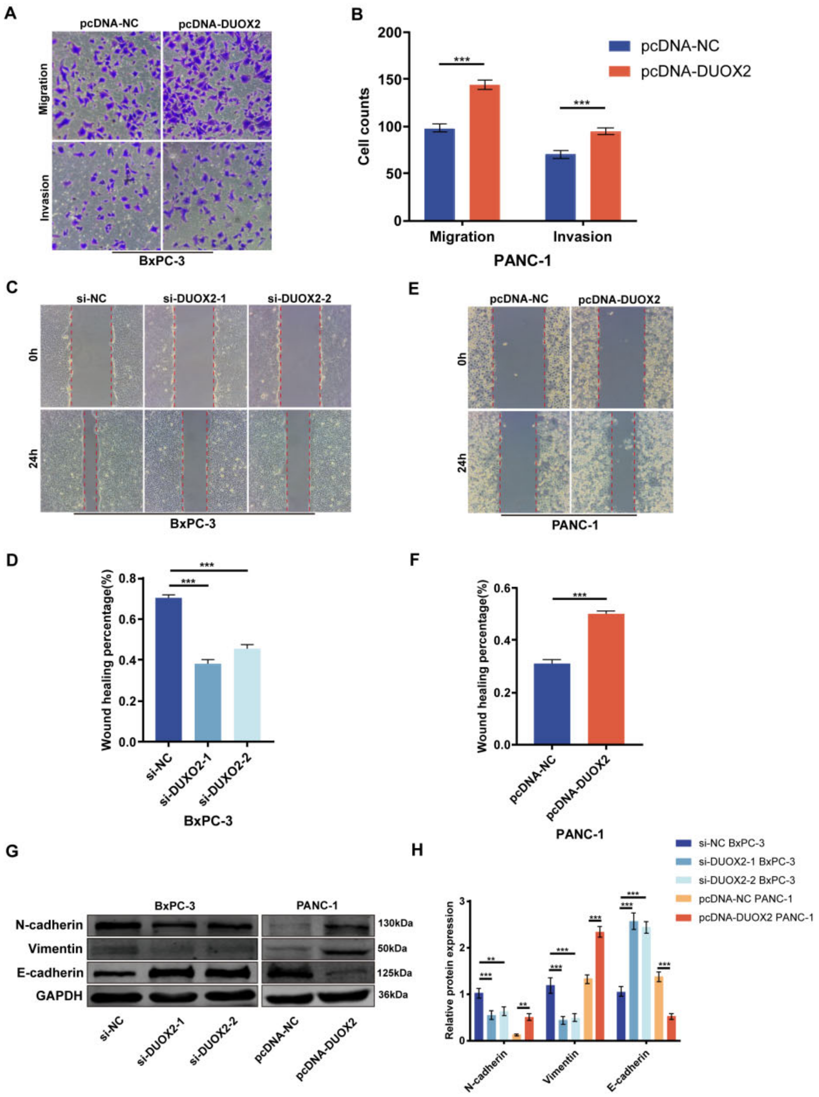

(A, B) Transwell migration/invasion assays assessing the effect of DUOX2

overexpression on invasive and metastatic capacities. (C–F) Wound-healing assays evaluating the migratory ability of pancreatic cancer cells following DUOX2 modulation. (G, H) Western blot analysis of EMT markers (N-cadherin, vimentin, and E-cadherin) after DUOX2 knockdown or overexpression. Three independent experiments were performed. Data is displayed as the mean ± SD. ns: no significance; *$P < 0.05$; **$P < 0.01$; ***$P < 0.001$.

Supplementary Figure S6. TCN1 regulates pancreatic cancer cell proliferation through DUOX2.

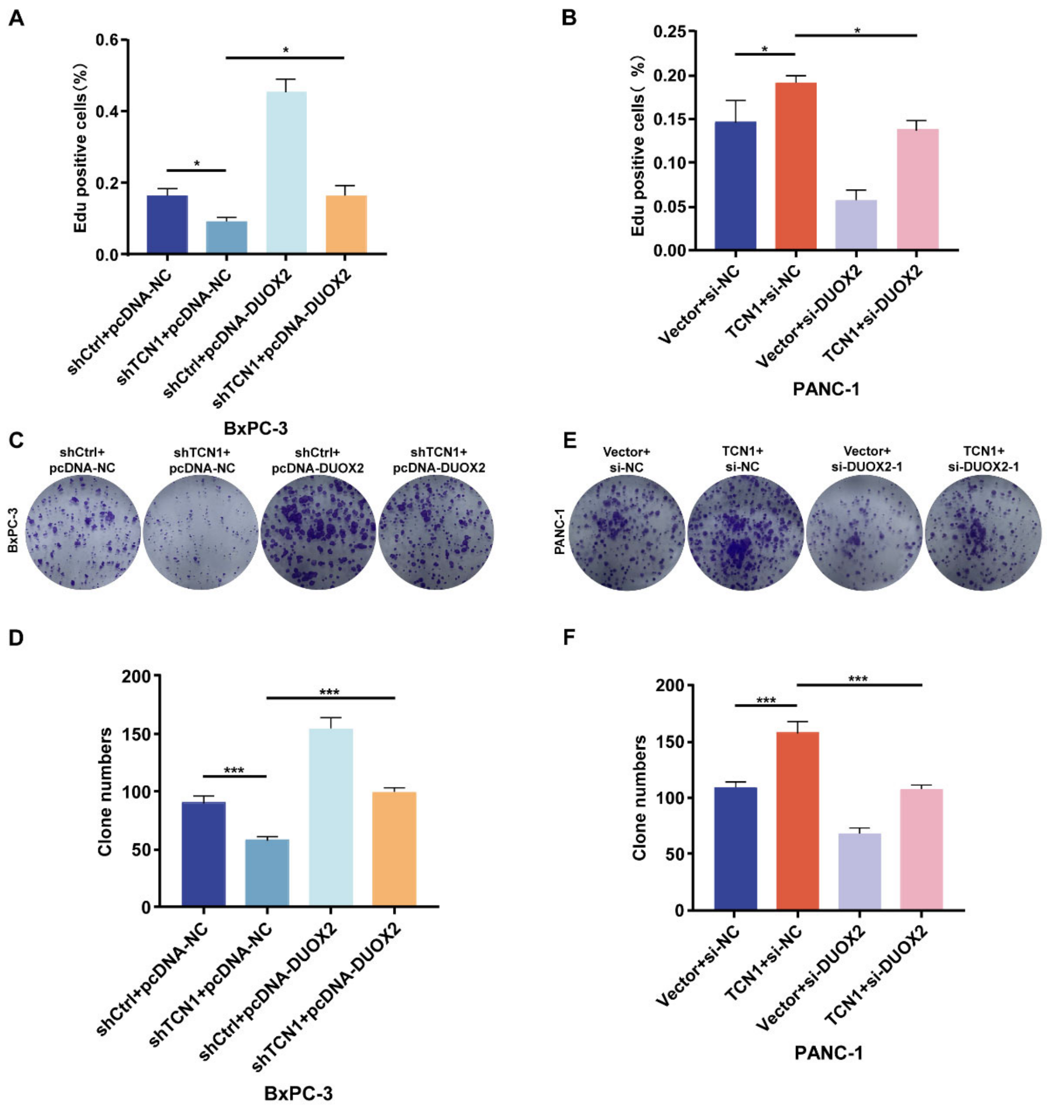

(A, B) EdU assay quantifying the rescue effect of DUOX2 modulation on TCN1-mediated changes in proliferation. (C–F) Colony formation assays showing the rescue of TCN1-driven proliferative effects by DUOX2 knockdown or overexpression. Three independent experiments were performed. Data is displayed as the mean ± SD. ns: no significance; *P < 0.05; **P < 0.01; ***P < 0.001.

Supplementary Figure S7. TCN1 modulates invasion and metastasis through DUOX2.

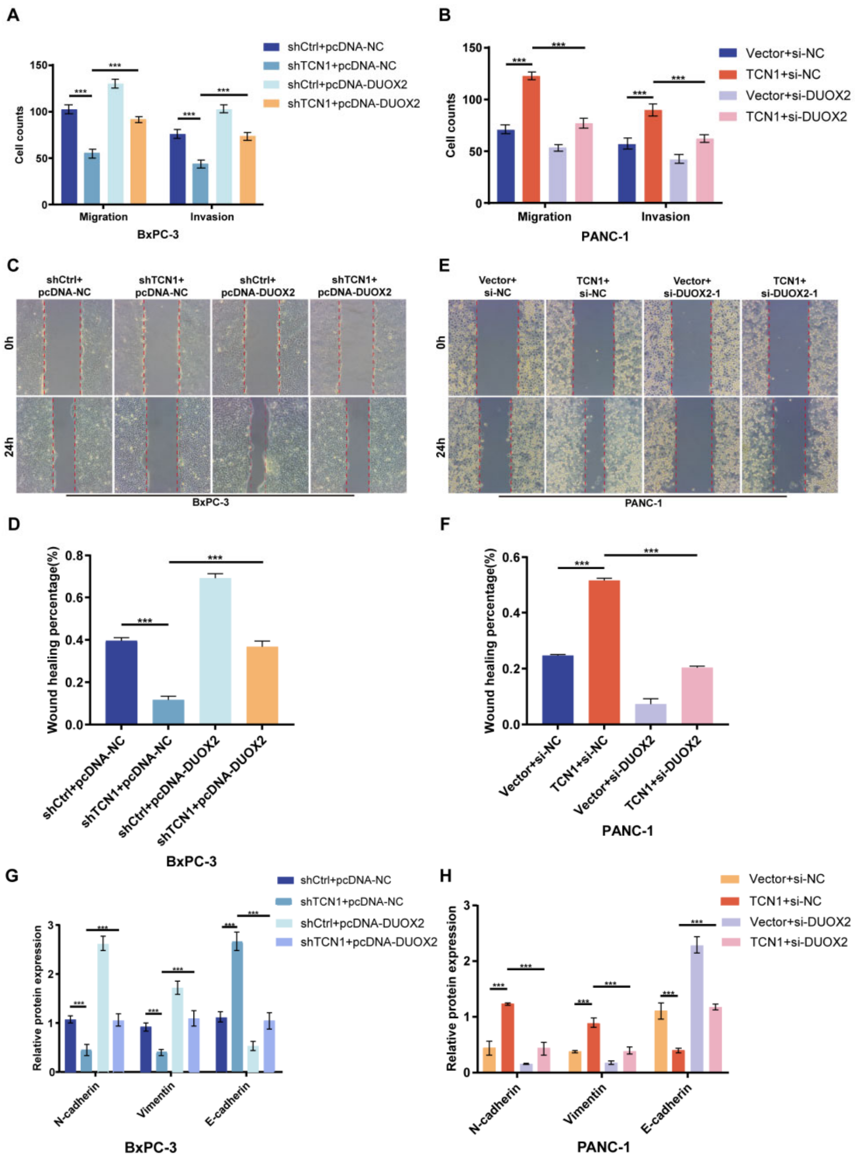

(A, B) Quantitative analysis of Transwell assays evaluating the restoration of TCN1-driven invasive/metastatic capacities through DUOX2 modulation. (C–F) Wound-healing assays showing the reversal of TCN1-induced migratory/invasive phenotypes through DUOX2 modulation.(G, H) Western blot densitometry of EMT markers (N-cadherin, vimentin, E-cadherin) under the indicated rescue conditions in BxPC-3 and PANC-1.Three independent experiments were performed. Data is displayed as the mean ± SD. ns: no significance; *P < 0.05; **P < 0.01; ***P < 0.001.

Supplementary Figure S8. Impact of TCN1 modulation on STAT4 transcription and phosphorylation in PDAC cells.

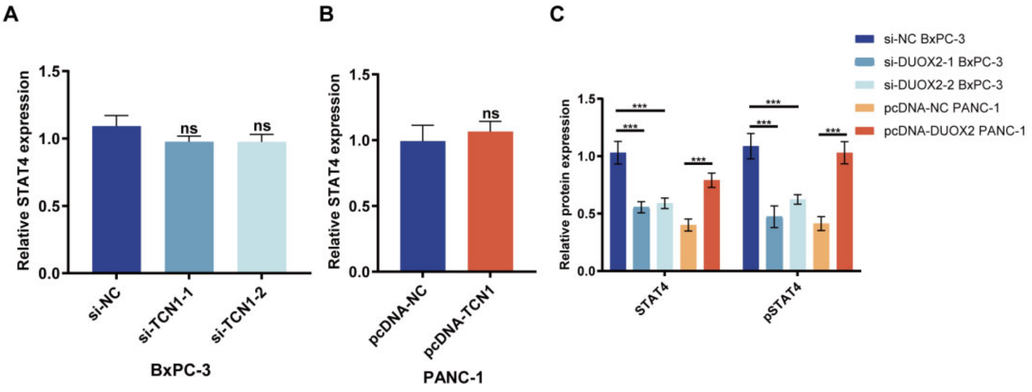

(A, B) qRT-PCR analysis of STAT4 mRNA after TCN1 perturbation (C) Western blot densitometry showing total STAT4 and phosphorylated STAT4 (p-STAT4) following TCN1 knockdown or overexpression in BxPC-3 and PANC-1 Three independent experiments were performed. Data is displayed as the mean ± SD. ns: no significance; *P < 0.05; **P < 0.01; ***P < 0.001.

Supplementary Figure S9. TCN1 regulates proliferation through STAT4.

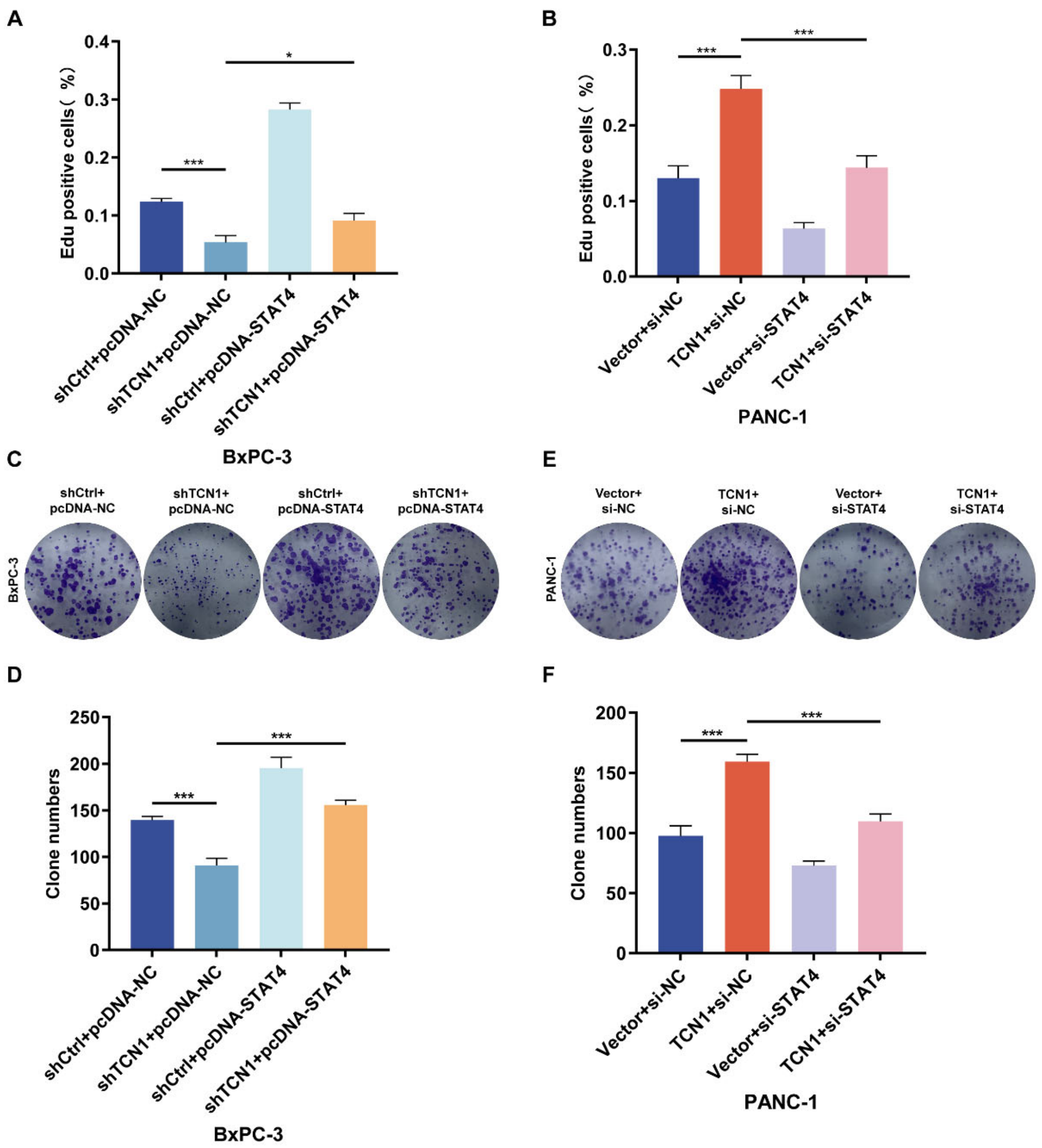

(A, B) EdU assay quantifying the rescue effect of STAT4 modulation on TCN1-mediated proliferation. (C, D) Colony formation assays assessing the rescue of TCN1-driven proliferation by STAT4 knockdown or overexpression. Three independent experiments were performed. Data is displayed as the mean ± SD. ns: no significance; *P < 0.05; **P < 0.01; ***P < 0.001.

Supplementary Figure S10. TCN1 alters invasive/metastatic behavior through STAT4.

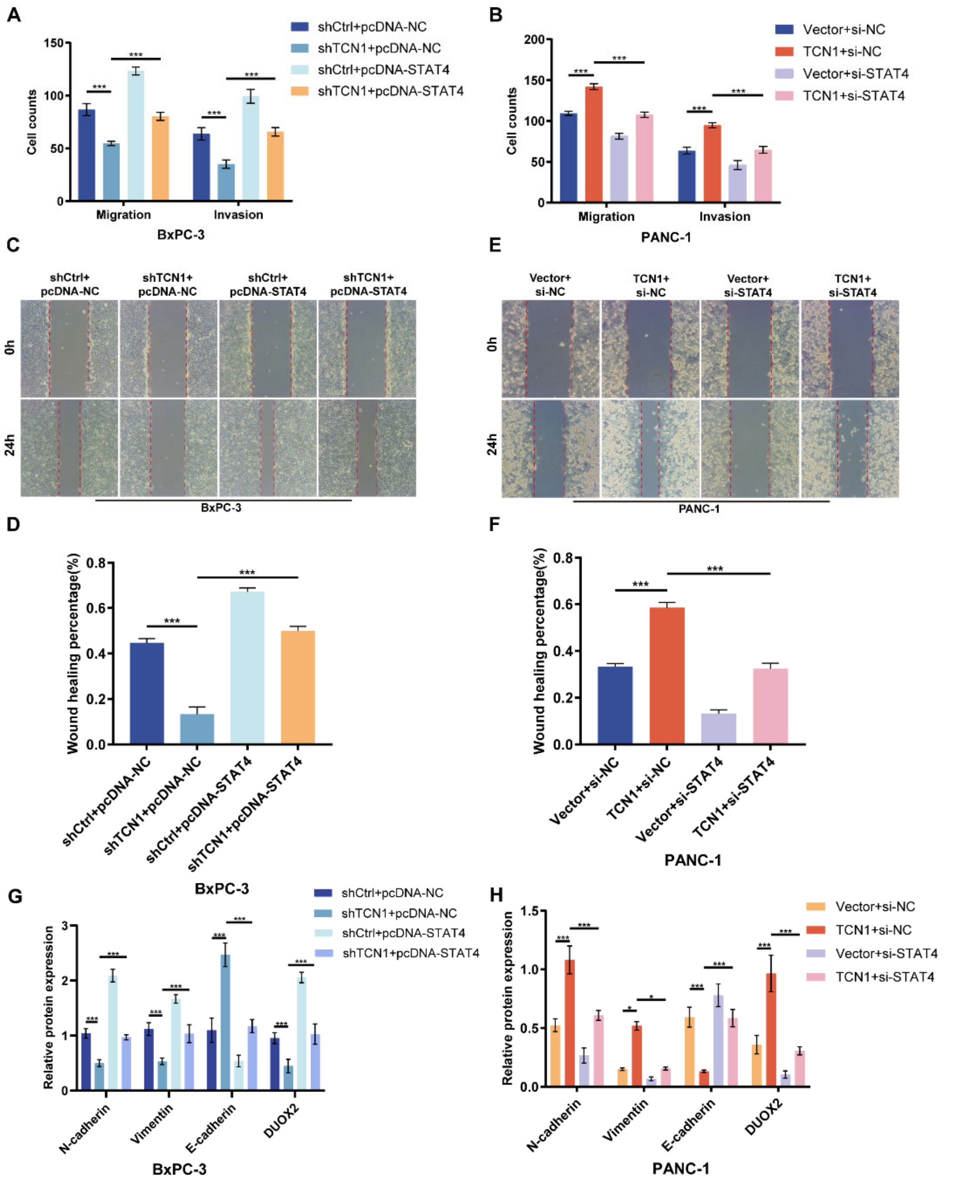

(A, B) Transwell assays quantifying the rescue of TCN1-driven invasion/metastasis through STAT4 modulation. (C–F) Wound-healing assays showing the reversal of TCN1-induced migratory/invasive capacities through STAT4 modulation. (G, H) Western blot densitometry of EMT markers and DUOX2 under the indicated rescue conditions in BxPC-3 and PANC-1. Three independent experiments were performed. Data is displayed as the mean ± SD. ns: no significance; *P < 0.05; **P < 0.01; ***P < 0.001.

Supplementary Figure S11. TCN1 drives malignant phenotypes through ROS.

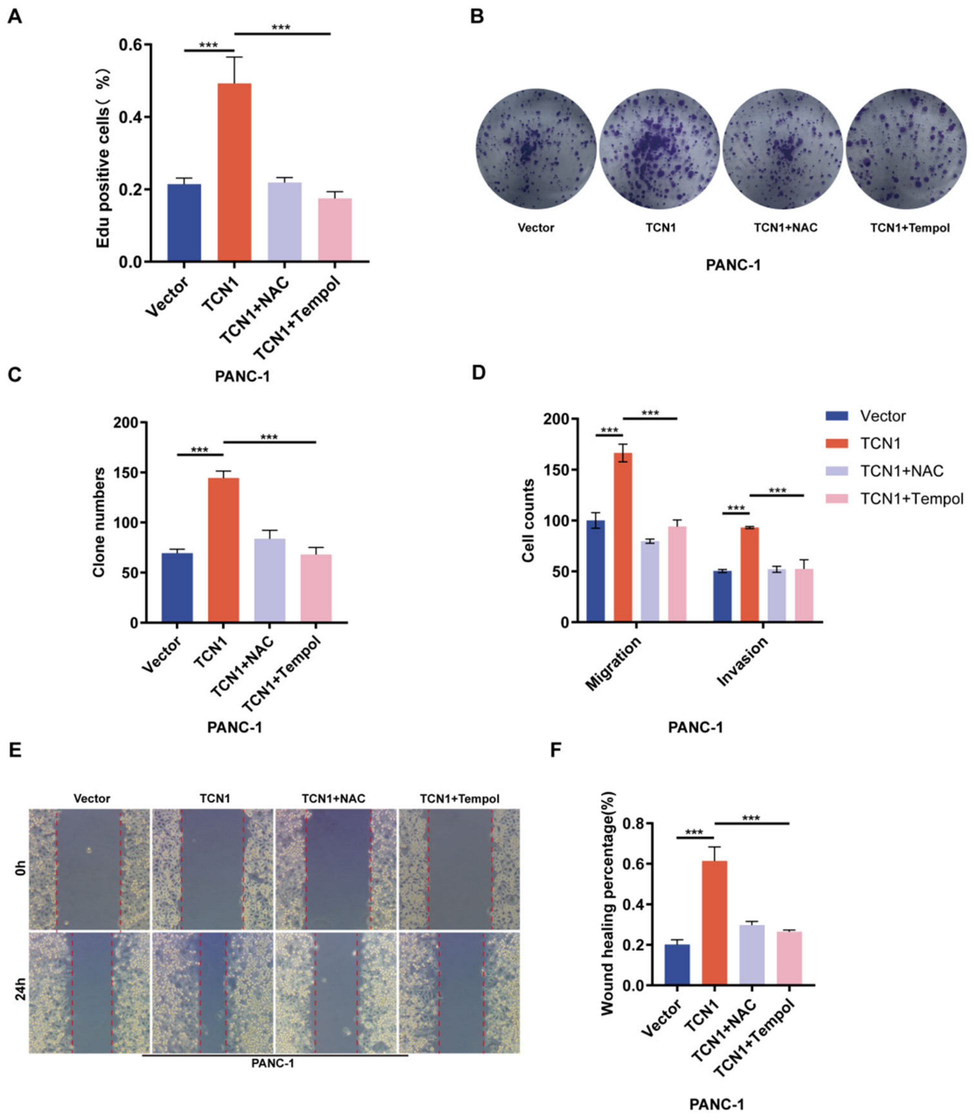

(A) EdU assay evaluating the reversal of TCN1-driven proliferation by the antioxidant N-acetylcysteine (NAC) and 4-Hydroxy Tempo (Tempol). (B, C) Colony formation assays demonstrating that NAC and Tempol suppress TCN1-induced clonogenic growth. (D) Transwell assay quantifying the rescue of TCN1-driven invasion/metastasis by NAC and Tempol. (E, F) Wound-healing assays confirming the ability of NAC and Tempol to reverse TCN1-induced migratory/invasive capacities.

Three independent experiments were performed. Data is displayed as the mean ± SD.

ns: no significance; *P < 0.05; **P < 0.01; ***P < 0.001.
